# NLP-Based Approach for Predicting HMI State Sequences Towards Monitoring Operator Situational Awareness

**DOI:** 10.3390/s20113228

**Published:** 2020-06-05

**Authors:** Harsh V. P. Singh, Qusay H. Mahmoud

**Affiliations:** 1 Department of Electrical, Computer and Software Engineering, Ontario Tech University, Oshawa, ON L1G 0C5, Canada; qusay.mahmoud@ontariotechu.ca; 2 Computers, Controls and Design Department, Ontario Power Generation, Pickering, ON L1W 3J2, Canada

**Keywords:** Human Machine Interface (HMI), human-in-the-loop (HITL), natural language processing (NLP), situational awareness (SA), sequence-to-sequence (seq2seq)

## Abstract

A novel approach presented herein transforms the Human Machine Interface (HMI) states, as a pattern of visual feedback states that encompass both operator actions and process states, from a multi-variate time-series to a natural language processing (NLP) modeling domain. The goal of this approach is to predict operator response patterns for n−ahead time-step window given k−lagged past HMI state patterns. The NLP approach offers the possibility of encoding (semantic) contextual relations within HMI state patterns. Towards which, a technique for framing raw HMI data for supervised training using sequence-to-sequence (*seq2seq*) deep-learning machine translation algorithms is presented. In addition, a custom *Seq2Seq* convolutional neural network (CNN) NLP model based on current state-of-the-art design elements such as attention, is compared against a standard recurrent neural network (RNN) based NLP model. Results demonstrate comparable effectiveness of both the designs of NLP models evaluated for modeling HMI states. RNN NLP models showed higher (≈26%) forecast accuracy, in general for both in-sample and out-of-sample test datasets. However, custom CNN NLP model showed higher (≈53%) validation accuracy indicative of less over-fitting with the same amount of available training data. The real-world application of the proposed NLP modeling of industrial HMIs, such as in power generating stations control rooms, aviation (cockpits), and so forth, is towards the realization of a non-intrusive operator situational awareness monitoring framework through prediction of HMI states.

## 1. Introduction

Several severe industrial and aviation accidents have brought grave lessons around minimizing operator error, strengthening lax safety culture, and improving Human Machine Interface (HMI) system designs. Previous nuclear power (NPP) accidents such as Three Mile Island (TMI) NPP, USA, and Chernobyl NPP disaster, USSR, have been classified high on the severity rating (ranging between 5 to 7) IAEA International Nuclear Event Scale (INES) [1]. At TMI (INES-5), poorly designed ambiguous control room indicators introduced operator error to override the emergency cooling water supply causing a partial meltdown of Unit 2 (TMI-2) reactor core containment on 28 March 1979. In the Chernobyl disaster, USSR (INES-7), where confounding human factors and inherent design flaws led to a catastrophic reactor Unit 4 explosion and release of radioactivity on 26 April 1986. Efficacy of such adverse event(s) bear tidings of compromised command inputs by highly trained control room nuclear operators (CNOs) owing to inaccurate cognitive model of dynamic unit evolutions and normalization to deviance to poor nuclear safety culture. Similarly, recent aviation industry accidents, such as Lion Air Flight 610 outbound from Jakarta, Indonesia, crashed on 29 October 2018. The root cause was attributed to a single point vulnerability in the last design update, associated with a single malfunctioning sensor on the Boeing 737 Max 10. This flaw falsely triggered the Manoeuvring Characteristics Augmentation System (MCAS) system. A failure mode unbeknownst to its pilots, repeatedly pushed the aircraft’s nose down, causing it to crash tragically.

Such accidents indicate a common theme of confounding factors that challenge human operator performance owing to human factor engineering-related design flaws in the HMI system. Key accident precursors as evident from post-accident reports [2,3] also reveal—(1) reduction in situational awareness owing to human factors related deficiencies in legacy HMI design; (2) normalization to deviance to lax nuclear safety culture; (3) information overload (looking-but-not-seeing effects [4]) owing to the rapid rate at which information was presented to CNOs via the control room HMIs (panel indications, annunciations, etc.); and (4) incorrect mental model of highly dynamic unit evolutions resulting in cognitive errors, owing to conflicting plant information supplied by failed or faulty sensors, as some of the root-causes of such accidents.

**Figure 1:**
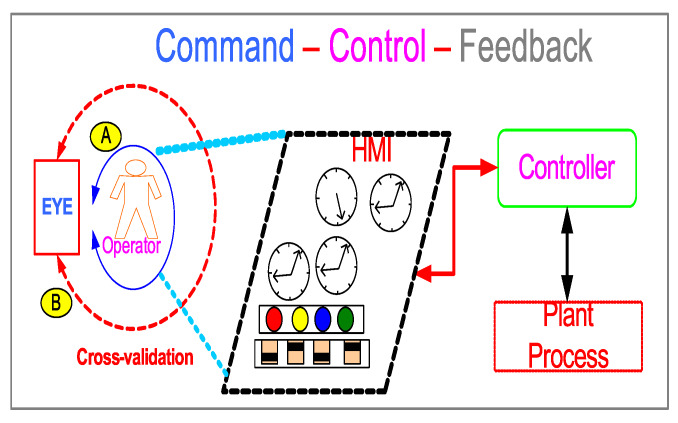
Command-Control-Feedback architecture is ubiquitous to most industrial and transportation operations. (**A**) The operator in the control loop visually acquires most plant process information via Human Machine Interface (HMI) states, perform required command actions and receive feedback via the displayed HMI states. (**B**) The novel EYE-on-HMI [5] system framework addresses cross-validation of visual feedback (HMI states) against expected operator actions and process information. Thereby achieving an independent and non-intrusive supervisory monitoring system in order to detect human-in-the-loop error precursors.

CNOs and pilots must rely on manual effort and acquired cognitive skills to overcome the fundamental limitation inherent in the conventional operator based command-control-feedback architecture (Figure 1A), vis-á-vis errors injected by human command inputs via HMIs either due to reduction in situational awareness or misinformation being displayed by the HMI due to some malfunction. While rigorous operator training does minimize human command input errors, in reality, system faults continue to fatigue the human brain owing to sensory overload thus, increasing chances of human-in-the-loop cognitive errors.

The above challenge is addressed by the previously proposed EYE-on-HMI [5] framework (EYE: **E**xpert supervisor**Y** syst**E**m framework for industrial control room HMIs.), which integrates a closed-loop independent cross-validation supervisory checks in the conventional operator based command—control—feedback architecture (Figure 1B). Cross-validation can be achieved by verifying what operator is visualizing (HMI visual feedback) on the HMI, truly matches the plant process state. Closed-loop logging and verifying operator command input patterns in response to the current HMI state closes the loop between process and operator actions. EYE-on-HMI proposes to achieve this by incorporating machine-learned models to predict the most likely sequence of operator actions *n-steps* ahead into the future, based on process and HMI state patterns at few steps in the past.

Several unique solutions may be possible to realize a system based on this proposed framework. However, the main goal of this work is to introduce a Natural Language Processing (NLP) modeling approach as a more generalized modeling technique over the previously proposed multi-variate time-series modeling techniques [6]. The objective is to learn both operator action patterns in conjunction with HMI state patterns (outputs). Predicting both HMI states and corresponding operator actions is a vital step towards achieving the primary objective of the proposed EYE-on-HMI framework, towards monitoring situational awareness in real-time, as discussed above.

For instance, if the predicted HMI output is different from the actual future HMI state patterns, it may be the reason to expect a deviation in predicted operator response. Conversely, if the predicted HMI output aligns with actual process states, but the predicted operator inputs vary, it may indicate a potential anomaly to be alarmed (addressed as future work). The challenge lies in modeling the HMI state patterns, including complex contextual dependencies between various HMI state features (parameters) to predict HMI states (including operator actions) accurately.

*Why explore NLP using machine translation (MT)?* Our motivation is based on the very nature of NLP, which makes it inherently non-deterministic and computationally *NP-hard*. Nevertheless, it also yields *“interesting”* machine translation algorithms that attempt to encompass several nuances of natural language translation more effectively. These include (but not limited) working with large dictionary of word spaces, morphology (word forms), syntax (grammar), and pragmatics (context-based) rules. MT deep learning algorithms offer a more generalized approach over multi-variate time-series anomaly detection models, despite recent advancement in using attention context for improving temporal patterns [7] forecasting. HMI visual feedback states are mixed mode processes consisting of both temporal and stateful parameters, hence requiring a modeling technique that is both expressive and scalable. Expressive model, refers to capturing complex multi-dimensional HMI indication and operator action pattern interactions in the context of several plant process parameters (scalable). Therefore, the proposed application of NLP MT (deep-learning) models to capture effectively complex HMI state transitions for gauging operator situational awareness and predicting human-in-the-loop (HITL) errors is novel.

The paper is organized as follows: Section 2 provides background and related work review of Situational Awareness monitoring and state-of-the-art works for NLP using machine translation algorithms. Section 3 presents a systematic problem formulation. Section 4 outlines design aspects of the proposed and other NLP models evaluated herein. Section 5 discusses the experimental setup for evaluating the model prototypes. Section 6 provides a discussion of results. Finally, Section 7 concludes the paper and offers future research directions.

## 2. Background and Related Work

### 2.1. Situational Awareness

Situational awareness (SA) generally refers to the level of alignment of the human operator’s cognitive state with actual process states. Modeling situational awareness is an active area of research in cognitive sciences. Operator mental awareness of the current process state and being able to anticipate future process states based on past experience and training is the highest SA an operator can possibly maintain at all times. Numerous SA cognitive models have been suggested from the widely accepted three-level *Endsley* model [8] (perception, comprehension, and projection stages of SA), to more recent Holistic framework [9] and Causal model [10]. However, as per *Endsley* model, SA is related to the task workload, information rate, system design, and complexity of tasks, requiring multivariate analysis.

Notably, the level of automation in HMIs also affects operator SA in a systemic fashion. For instance, in the automation and situational awareness report [11] enlisted are several aviation accidents that were caused due to accidental failure of legacy automated systems which pilots had come to rely on. These events highlight the concern with an inevitable increase in the level of automation in complex HMI systems. Furthermore, this conjecture can conservatively be assumed relevant to modern automation design approaches, where human operators are increasingly being placed out of the loop (such as in autonomous or unmanned operations). It [11] states—“In examining these failures, it becomes apparent that the coupling of human and machine in the form of observer and performer is far from perfect in terms of optimizing the overall functioning of the joint human-machine system”. Therefore, a monitoring system that independently monitors the interaction between human operators and HMIs would be a definite improvement in catching human errors in complex automated HMI systems.

Previous techniques for monitoring SA, as addressed by various works [12], recognize it to be a multivariate data analysis challenge. As notably indicated by *Endsley* model [8], SA may be measured using objective measures, often requiring intrusive monitoring of operator physiological parameters (e.g., eye-movement tracking, frequent real-time queries, self-rating, etc.) or subjective measures via operator questionnaires when an event scenario is frozen during training. In addition, former objective techniques (e.g., SAGAT, SART [13,14] scores) that offer a real-time measure of SA by comparing the operators current cognitive state to an expected normal state, does so via intrusive monitoring which is quite burdensome. Therefore, using objective monitoring as the basis of monitoring SA, the proposed approach of EYE-on-HMI [5] rather relies on a non-intrusive monitoring technique by modeling HMI indication patterns encompassing operator response sequence.

### 2.2. Validation Using Naturalistic Data

The previously proposed EYE-on-HMI [5] framework relies on collecting naturalistic trial data available as visual feedback from industrial HMI control panels. This data may be collected non-intrusively via cameras pointed towards operator control panels, in order to ultimately model operator and HMI interaction patterns under various real-plant scenarios in an operator training simulator. This approach aligns with the Naturalistic Driving (ND) data collected using unobtrusive sensors to reliably collect human driver pattern data while operating vehicles in real-world driving scenarios. The resulting high-fidelity big data is an example of naturalistic experimentation. For example, driver visual glance patterns at signalized and unsignalized intersections [15] and difference in driver cognitive attention behaviour during traffic congestion and post-congestion driving scenarios [16], based on eye movement tracking and brain EEG signal data. Validation using this data has shown to reliably model operator behaviour patterns, as shown by Strategic Highway Research Program (SHRP 2) [17], modeling driver lane-changing behaviour in rainy weather [18]. However, the challenge remains to effectively utilizing this big data without necessarily curtailing its features, such as suggested by mapping this data using geographic information system [19]. Furthermore, ND aids in correctly characterizing the false-positive and false-negative behaviour of the model. The proposed NLP based HMI modeling approach is poised to address this challenge using non-intrusive naturalistic trial HMI data. The adopted modeling technique draws on the concepts from distributional encoding and attention mechanisms of language translation algorithms, as discussed below.

### 2.3. Word Embedding for HMI State Encoding

Natural Language Processing (NLP) using Machine Translation (MT) is a multidisciplinary and an active area of research under computational linguistics having several applications spanning engineering and business domains such as gene mapping, video/image analysis (image captioning), sentiment analysis, conversational agents (chatbots), market intelligence surveys, and so forth. NLP MT algorithms generally depend on key supportive technologies: word embedding, attention mechanism, and deep learning models [20]. A leap forward from traditional statistical NLP tasks to the current use of deep learning models was made possible by efficient word embedding techniques. Overcoming the *curse of dimensionality* in NLP while learning joint probability models for modeling complex language models was essential.

Word embedding architectures such as *CBOW*(continuous bag of words) and *skip-gram* have been used by *word2vec* [21], *GloVe*(Global Vectors for word representation) [22] algorithms, and so forth, to generate lower-dimensional denser vector representation of words using shallow feed-forward neural network. These embeddings take advantage of distributional semantic [23,24,25] similarity between the words in a given set of corpus space to produce a vector space, where words with similar meanings are located in closer proximity to yield a lower cosine (dot product) similarity score.

In addition to distributional semantics in word embeddings, further enrichment is achieved by incorporating additional context-specific information, which has been demonstrated by other works [26,27] to be useful in further improving NLP predictions. The limitations of the word-level embeddings (e.g., word2Vec) is that these do not quite produce vector representations of special word sequences or phrases which mean something more than just the words put together (*polysemy*), for example, “brown out,” “phase in,” and so forth. The latter is relevant to natural language translation tasks and may be useful for modeling complex context dependencies in system state patterns, (e.g., system state sequence S1→S2 could invoke different user response in the presence of another context state S3).

In the current scope of this work, due to a relatively smaller vocabulary size of HMI state space as compared to that encountered in human NLP tasks, existing word embedding was not utilized in order to keep the initial model design simpler. Nevertheless, distributional encoding shall be a vital consideration when the controller needs to scale up to more complex industrial HMI designs. Moreover, positional encoding in the proposed model is implicit by constructing the training data set using a shifted sliding window (described in detail in below Section 5.3).

### 2.4. Attention for NLP Models

**Figure 2:**
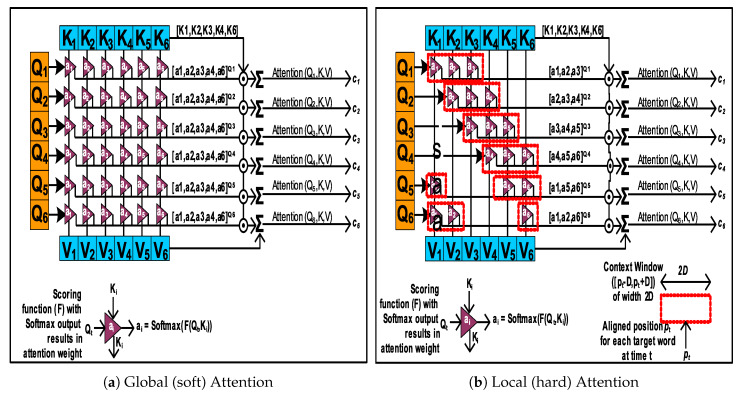
(**a**) Generates variable length (same as input sequence) context vectors $\left[c_{1},c_{2}..,c_{6}\right]$ which capture attention weights ($\left[a_{1},a_{2}..,a_{6}\right]$) from all input tokens (**K**); (**b**) Generates a fixed length context vector $\left[c_{1},c_{2}..,c_{6}\right]$ which capture fixed number of attention weights ($\left[a_{1},a_{2},a_{3}\right]$, etc.) from select input tokens selected by a context window which is centred at token index position $p_{t}$ (determined by the model separately).

Attention mechanism was initially introduced in 2014, to build efficient computer vision algorithms, mimicking human vision. It works by learning to use selective concentration on smaller parts of a larger scene to derive features [28] for recurrent neural network (RNN) based image classification. However, first applications of AM in NLP (machine translation) tasks did not come about until early 2015 [29,30], where *global* (Figure 2a) and *local* (Figure 2b) AM types were first introduced. The *global* AM approach uses contribution from all source tokens (*encoder* hidden states) in jointly learning to align the *decoder* (hidden state) to produce an output token. On the other hand, the *local* AM uses limited source tokens in order to reduce training computational load. Attention mechanism (AM) (Figure 10 described in Section 4.1.3) has been used to over come the information bottleneck in classic *encoder-decoder* models (Figure 7) for NLP *seq2seq* applications.

AM has significantly improved NLP performance for *alignment* and *translation* tasks: *alignment* refers to learning parts of the input sequence that are relevant to each output target token and *translation* is learning to use relevant contextual information between source tokens to select the appropriate output token. This overcomes the information bottleneck in classic *encoder-decoder* models (Figure 7) for NLP *seq2seq* applications.

The *encoder* extracts the similarty between various input source tokens (Key or Value) and an expected output token (Query). The *decoder learns* through training, to selectively *attend* or focus on various positions in the input sequence based on the tokens predicted so far [29] in order to accurately predict the next token in target language.

Self-attention or intra-attention is also another specific case of AM which finds relationships between positions of a single sequence to compute a compressed feature representation of the same sequence. In essence self-attention is *Self-Attention(Q,K,V)* where, Q=K=V and means attention is applied to each token of the sequence [31] with other tokens of the same sequence. Self-attention has been used in image description and text sentiment analysis applicaitons [31]. However, self-attention layers can also be stacked together in both *encoder-decoder* models to aid in machine translation tasks as demonstrated by the *transformer* [26] model which is further discussed below. Self-attention is also unique in resulting only a constant (O(1)) longest path dependency between source tokens for any length of sequence, compared to other AM (e.g., linear O(n) for RNN *seq2seq* models). This effectively allows the model to focus on the semantic dependency relationships between the tokens rather than based on encoding distances between them.

### 2.5. NLP Models

#### 2.5.1. Transformer

**Figure 3:**
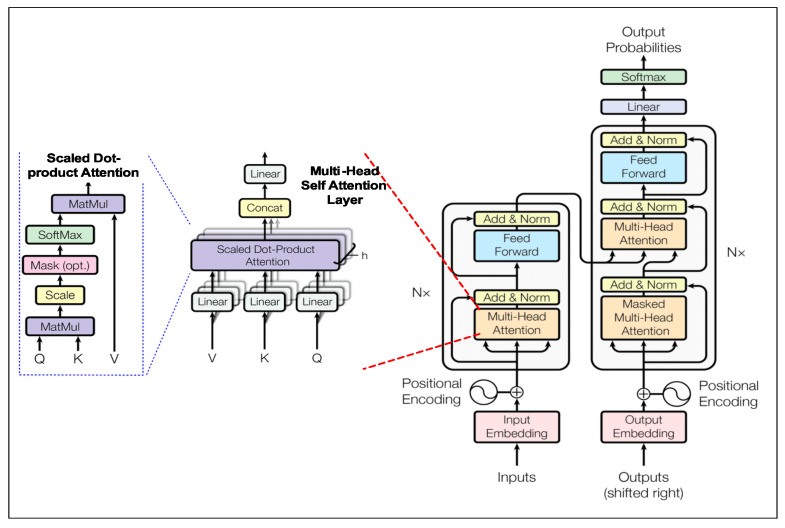
Transformer Model Architecture. Transformer Architecture [26] is parallelized for *seq2seq* applications which is neither CNN nor RNN based. Encoder-decoder branches consists of stacked multi-headed self-attention layers with scaled-dot product function.

Even though the *transformer* NLP *seq2seq* model has been analyzed in numerous works [32,33] previously, it is worth reviewing the architectural highlights in brevity due to its contribution of establishing a unique and dominant modeling paradigm in machine translation applications, since its introduction in 2017. The model (Figure 3) demonstrated a feasible alternative to existing RNN and CNN based *seq2seq* models to models for which authors claimed, *Attention is all you need*. It demonstrated using just a few stacked multi-headed self-attention layers with fully connected dense layers to achieve state-of-the-art performance in NMT [32,34].

Key architectural highlights include, firstly a parallelized self-attention similarity function: the Scaled-dot product (Figure 3). It first calculates similarity score using inner product between each (either *encoder* or *decoder*) hidden state vectors ***Q*** ($\left[q_{1},q_{2},\ldots ,q_{n}\right]$) against all other hidden state **Key** or **Value** pairs ($\left[\left(k_{1},v_{1}\right),\left(k_{2},v_{2}\right),\ldots ,\left(k_{m},v_{m}\right)\right]$) of the same sequence (K=V): $K^{⊤}$. The resulting matrix is then scaled with $\sqrt{d_{k}}$, where $d_{k}$ is the dimensionality of the **Key** (source or target token) word embedding, that prevents the inner product to become too large (1). Followed by application of *softmax* operation to normalize the 2d (n×m) similarity score matrix resulting in the attention weight matrix of same shape. Lastly, an inner product of the attention weight matrix is performed with the Value vector ($m\times d_{k}$). This effectively results in a 2d matrix ($n\times d_{k}$) containing weighted sum of all **Values** for each **Query** ($Q_{t}$), where the weight assigned to each **Value** ($V_{i}$) is the attention weight.

(1) $$\begin{array}{c} \begin{array}{c} \mathrm{Scaled}-\mathrm{Dot}\;\mathrm{Product}\\ Attention\left(Q,K,V\right)=softmax\left(\frac{QK^{⊤}}{\sqrt{d_{k}}}\right)V, \end{array} \end{array}$$

where:

*m,n*—source and target sequence length containing words (tokens) respectively,
$d_{k}$—word (token or state) embedding dimension,
$Q\in R^{n\times d_{k}}$—Query (decoder input) target sequence hidden vector shape,
$K\in R^{m\times d_{k}}$, $V\in R^{m\times d_{k}}$—Key/Value (encoder input) source sequence hidden vector shape.

Secondly, it uses N=6 parallel stacks of multi-headed self-attention Equation (2) sub-layers for both *encoder* and *decoder* branches of the model (Figure 3). Each layer computes *h* different linear transformation of (*Q,K,V*) controlled by a learned parameter *W*. Each representation of *Q,K,V* go through *h* scaled-dot attention computations, each of which is referred to as an attention *head*, thus its called *h-headed* self attention. Finally, all *h* iterations of the Scaled-dot attention *heads* are concatenated and fed to another parametrized ($W^{O}$) linear transformation to output the final context vector (*Z*).

(2) $$\begin{array}{c} \begin{array}{c} \mathrm{Transformer}\;\mathrm{Multihead}\;\mathrm{Self}-\mathrm{Attention}\\ MultiHead\left(Q,K,V\right)=\left[head_{1};\ldots ;head_{h}\right]W^{O}, \end{array} \end{array}$$

where:

$head_{i}$ = $Attention(QW_{i}^{Q},KW_{i}^{K},VW_{i}^{V})$,
$W_{i}^{Q},W_{i}^{K},W_{i}^{V},\&,W_{i}^{O}$—are parameter matrices to be learned.

Each *transformer encoder* and *decoder* attention layer combines by addition of self-attention output *Z* with the original positional encoded input sequence (*Q*) and normalizes the results. The addition operation is similar to adding residual connections within each layer to reduce over-fitting and learning saturation. The output of *add and norm* operation is then fed to a feedforward (FF) neural network (auto-encoder), which re-shapes the output in the desired dimension for a yet another final *add and norm* operation.

The only difference between *transformer encoder* and *decoder* branches is the latter includes an additional multi-headed self-attention sub-layer, which merges the *decoder* input and *encoder* input attention based representations together as input to the subsequent multi-headed self-attention sub-layer in *decoder*. Final *transformer* output is simply a linear dense layer followed by *softmax* operation to emit the target token class probabilities.

#### 2.5.2. BERT and SNAIL

Other notable model architectures that have extended the basic *transformer* architectures are: BERT and SNAIL.

BERT [35] (Bidirectional Encoder Representations from Transformers) has shown to achieve (2018) state-of-the-art performance for various NLP tasks such as in question answering (SQuAD v1.1 database) [36], natural language inference (NLI) [37]. This architecture utilizes the *transformer* encoder to allow transfer learning. In that, it demonstrated developing a pre-trained deep NLP model, which can be fine-tuned with the addition of just one output layer to customize to a various range of applications without any added training overhead compared to other existing transfer learned models [38]. Furthermore, BERT adopts using a non-directional language learning model, unlike other NLP models that learn sequences sequentially (either left-to-right or right-to-left), thereby proposing two learning regimes: Masked Language Model (MLM) and Next Sentence Prediction(NSP). This allows BERT to easily be fine-tuned as its pre-trained with the learning goal to not just predict the next token in sequence, but learning the token-level context from both direction crucial to question answering tasks.

Even though *transformer* model has shifted away from using RNN and CNN for capturing inter-sequence dependencies, despite it using positional encoding to retain the sequence information. *Transformer* model may sometimes fall short for applications where a very long sequence of tokens exist, such as in meta-token sequence learning are required.

The SNAIL (Simple Neural Attentive MetaLearner) [39] architecture prioritizes capturing the sequential information in sequences by using temporal convolutions with causal attention layers. This is in contrast to*transformer* architecture that can draw context relationships from infinitely long sequences due to self-attention in which *Query* and *Key-Value* pair, are treated as unordered tuples lacking positional dependence. This can be undesirable, especially for reinforcement learning, where the observations, actions, and rewards are intrinsically sequential [39].

## 3. Problem Formulation

The initial problem at hand task is to (1) establish a theoretical model for generic HMI indication sequences that is sufficient to capture both HMI information and user actions (2) identify the key assumptions and limitations of this generic HMI model (3) how this generic HMI model can be transformed into a model conducive for NLP using machine translation techniques.

Based on the above, an HMI state-space model is initially developed below to aid in generalizing time-series patterns generated by a typical HMI system. Such patterns are intended to capture both the indications and operator actions. Moreover, there are two key assumptions that enable us to formalize the proposed generic HMI state-space model, as discussed below.

The key challenges addressed herein are (1) Formally mapping the generic HMI state-space model (conducive for regression-based modeling) into a discrete event system (DES) model (conducive for NLP based modeling). (2) Demonstrate and evaluate the utility of machine translation deep learning models for the HMI DES model.

### 3.1. HMI State Space Features

**Figure 4:**
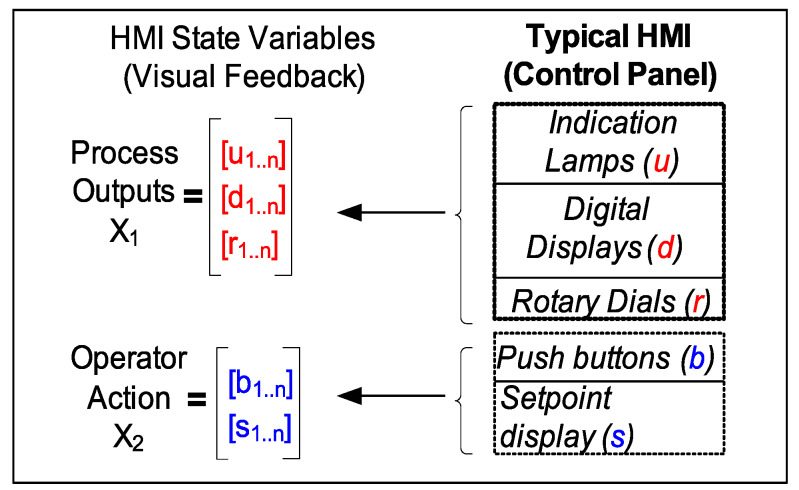
Conceptual data model for a typical industrial HMI (Control Panel), which is represented by two multidimensional feature vectors for Process outputs and Operator Action ($X_{1}$ and $X_{2}$). Process output for each output devices, for example, Indication lamps, digital displays, rotary dials states or values, corresponding to particular dimensions of $X_{1}$. Operator actions that are indicated by push button, hand switches or setpoint display states corresponding to particular dimensions of $X_{2}$. All above HMI states are available as visual feedback.

Conceptually, HMI states (Figure 4) for any typical plant process may be captured by two categories of state features: process output (*PROCESS*: $X_{1}$) and human input (*HMI_USER*: $X_{2}$) vector. Each feature vector variable can be a tuple of binary-valued states (e.g., indication lamp states, pushbuttons states, etc.) and/or a finite range of analogue valued states (e.g., rotary dial indicators, digital setpoint displays, etc.).

For example, in Figure 4, Process outputs: q1,q2,q3 are analogue variables associated with each rotary dial value and q4, a *4-bit* binary word, can capture indication patterns of all four lamp string. Similarly, Human operator inputs: i1,i2,i3,i4 as digital variables, can be associated with each push buttons and i5 unsigned integer variable can be associated with a setpoint indicator. An ensemble of such multi-type variables (or features) can sufficiently capture all states of the HMI. Trending the HMI state features yields multi-variate time series data.

### 3.2. HMI Model Assumptions

Two key assumptions for HMI state patterns to be modeled for time-series prediction are rationalized below.

Firstly, a HMI process may not yield a data series that is *white noise* (ε)—a series that is generated by random variables that are independent and identically distributed, that is, having zero mean (μ=0), with identical finite variance $\left(\sigma^{2}<\infty \right)$ that are serially uncorrelated $E[\varepsilon_{t}\varepsilon_{k}]=0$ for all t≠k Equation (3).

Otherwise, a HMI displaying white noise state patterns would suggest an error or malfunction in the external process displayed by the HMI system.

(3) $$\begin{array}{c} \begin{array}{c} \mathrm{White}\;\mathrm{Noise}\\ x_{t}=\varepsilon_{t}, \end{array} \end{array}$$

where:

$\varepsilon_{t}∼\left(0,\sigma^{2}\right)$ with $\sigma^{2}<\infty$, and $E[\varepsilon_{t}\varepsilon_{k}]=0$ for all t≠k.

**Figure 5:**
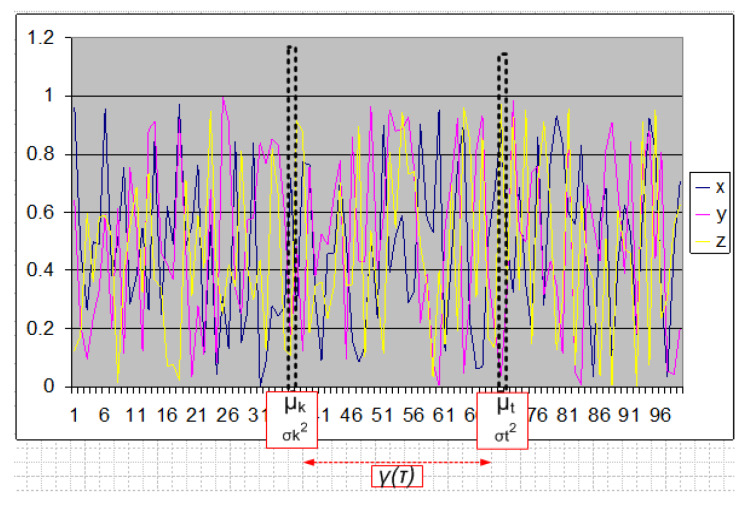
Depiction of an arbitrary Weakly stationary time-series traces (*X,Y,Z*) all generated from a stochastic process whose statistical properties are time invariant between various runs: implying the means ($\mu_{k}$, $\mu_{t}$) and variances ($\sigma_{k}^{2}$,$\sigma_{t}^{2}$) are finite and relatively constant over various time slices. Moreover, auto-covariance (γ(τ)) between various time slices is finite and is only a function of temporal distance τ=|t−k|.

The second assumption is that a majority of HMI state transition can be modeled as stochastic processes that yield a weak stationary (Figure 5) multi-variate time-series in most practical scenarios. A weakly stationary process must satisfy three conditions as listed in Equation (4) and yields a time-series (Figure 5) where (1) mean (μ<∞) (2) variance $\left(\sigma^{2}<\infty \right)$ are approximately finite and constants for all *t* windows (i.e., these do not vary with time), while (3) the auto-covariance (γ(t,k)) between any observed values at two time slices of a stochastic process is finite and constant for all τ, that is, the auto-covariance of a weakly stationary time series (TS) only depends on the temporal distance (τ=|t−k|) between any two time points (*t* and *k*). Auto-covariance, when normalized by the standard deviation of each observation of TS, results in auto-correlation function (ACF), which makes the measurement metric unitless. ACF Equation (5) is calculated for various lagged versions of the TS, which show the degree of similarity of TS with a lagged version of itself indicating the presence of various patterns that can be modeled linearly.

(4) $$\begin{array}{c} \begin{array}{c} \mathrm{Weakly}\;\mathrm{Stationary}\;\mathrm{Requirements}\\ \mu <\infty \;\mathrm{for}\;\mathrm{all}\;t,\\ \sigma^{2}<\infty \;\mathrm{for}\;\mathrm{all}\;t,\\ \gamma (t,k)=\gamma [\tau ]<\infty ,\;\mathrm{where}\;\tau =|t-k|,\;\mathrm{for}\;\mathrm{all}\;t\neq k. \end{array} \end{array}$$

(5) $$\begin{array}{c} \begin{array}{c} \mathrm{Auto}-\mathrm{Covarriance}\;\mathrm{and}\;\mathrm{Auto}-\mathrm{Correlation}\;\mathrm{Funtions}\\ \gamma \left(t,k\right)=Cov\left(X_{t},X_{k}\right)=E\left[\left(X_{t}-\mu_{t}\right)\left(X_{k}-\mu_{k}\right)\right],\\ ACF\left(\tau \right)=\rho \left(t,k\right)=Corr\left(X_{t},X_{k}\right)=\frac{\gamma (t,k)}{\sigma_{t}\sigma_{k}}, \end{array} \end{array}$$

where:

−1<ρ(t,k)<+1, for all t≠k.

The rationale supporting the above two assumptions is based on the fact that human-machine interfaces primarily display the process values and accept operator inputs as commands. The process parameter values are ultimately governed by underlying process control laws modeled by a system of differential equations that vary in a tight allowable band (range bounded). Moreover, the operator inputs also change in some correlation to the process values. Therefore, process values and operator inputs ought to display causality effects (i.e., either the process information having influence on operator actions or vice-versa). For most practical scenarios, the range of operator inputs is found not to vary indefinitely. Otherwise, those scenarios would require operator actions outside their normal range of trained behaviour (e.g., driving a vehicle on a freeway has set of rules every human driver normally adheres to). Hence, chances of any HMI state transition resembling that of a *random-walk* stochastic process is minimal, and therefore is not currently addressed in the scope of this work. Above, weakly stationary assumptions for the HMI generated time-series patterns make it possible to develop either linear regression-based forecast models based on ARIMA [6] or using non-linear recurrent networks such as LSTM.

### 3.3. HMI DES Modelling for NLP

Previously stated, weakly stationary assumption implies finite system state transitions, which allows treating the HMI system as a framework of a finite-automaton (FA) (either deterministic or non-deterministic). This model is broadly identified as a Discrete Event System (DES). DES, by definition, is an event-driven system where its state transition occurs with discrete events, and there is no restriction on the nature of state-space (Q) of a DES to be either discrete or continuous or mixed. Such basic qualities of DES aligns with the previously stated HMI model assumptions (Section 3.1).

**Figure 6:**
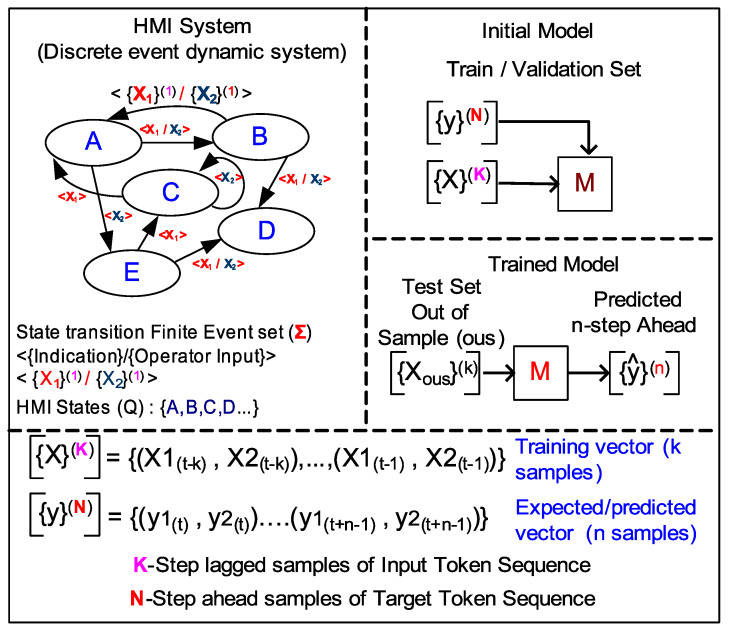
HMI System modelled as a Discrete Event System (DES).

Under the Ramadge and Wonham (RW) framework [40], a HMI DES plant model *P* can be obtained by a formal language generated by a “generator” FA (G), whose alphabet consists of the (finite) set of events (∑). The **generator** is defined using 5-tuple (6) parameters and can be depicted as a HMI DES directed graph (Figure 6) with its the nodes as DES states from Q set and edge set defined by pairs $\left(q,q^{\prime }\right)$, such that $\delta \left(q,\sigma \right)=q^{\prime }$ for some σϵ∑. That is, an edge between states *q* and $q^{\prime }$ can be labelled with event σ that transitions the HMI DES from state *q* to $q^{\prime }$.

The ∑ (6), is interpreted as the *alphabet* set corresponding to a finite set of **events** or directed edges in Figure 6, which maps to a particular value of a HMI feature vector $X=<\;X_{1},X_{2}\;>$ (Figure 4). The HMI DES state transition sequence is specified by δ, a partial function δ(q,σ) that is not required to be specified for all *q* (states) in Q and all σ (events) in ∑. In fact δ (6) is the function that must be learned and approximated by natural language processing (NLP) algorithm.

It is noteworthy to restate that RW framework only expects a finite set of HMI state transitions (∑) but not necessarily a finite HMI state (Q) set. This implies that the NLP model can be trained with a finite set of HMI events (*alphabet*) or dictionary of events, each corresponding to a point in HMI feature space *X*. A non-finite HMI state set implies HMI DES can have several sequence of events (s) or *strings*. This is the key realization that enabled us to consider evaluating NLP deep learning algorithm to model HMI DES.

Furthermore, HMI DES language model can be formally specified Equation (7) under RW framework as a **language** L(G) over an event set ∑, as any subset $\sum^{*}$ which captures all (finite) strings ***s*** built using elements in ∑.

(6) $$\begin{array}{c} \mathrm{HMI}\;\mathrm{DES}\;\mathrm{Model}\\ G=(Q,\sum ,\delta ,q_{0},Q_{m})\\ Q:\;\mathrm{States}\;\mathrm{set},\\ \sum :\;\mathrm{Events}\;\mathrm{set},\\ \delta (q,\sigma )=\sum \times Q\to Q, \end{array}$$

where:

qϵQ, σϵ∑,
$\left(q_{0},Q_{m}\right)$—(initial state, final marker state).

(7) $$\begin{array}{c} L\left(G\right)=\{s:s\;\epsilon \;\sum^{*}\subseteq \sum \;\&\;\delta \left(s,\sigma \right)\;\mathrm{is}\;\mathrm{defined}\} \end{array}$$

Lastly, HMI DES model under RW framework also inherently addresses scalability as DES can be expanded by incorporating sub-systems, or sub-processes $G_{1},\ldots ,G_{n}$ that are asynchronous and independent as long as each $G_{i}$ alphabet set $\sum_{i}$ are disjoint. That is the complete model of a HMI DES plant can be specified by **shuffling** the languages of HMI sub-systems (e.g., indication lamps, meters, pushbuttons, and so forth, with corresponding operator inputs) $L_{1}\ldots L_{n}$ which is denoted by $L_{1}\|L_{2}\left\|\ldots \right\|L_{n}$ and defined by Equation (8). Where, s↑i is the projection of **s** on $\sum_{i}$ that only keeps *alphabets* or events belonging to $\sum_{i}$. This implies, the complete HMI DES language model can be obtained by appending discrete event symbol and pattern sets from other HMI sub-systems.

In other words, **shuffling** Equation (8) property of HMI DES model addresses the scalability of the proposed NLP modelling approach by mapping favourably to current advancements [41] in *transfer learning* in the context of machine-learned NLP models. *Transfer learning* has the potential to use pre-trained robust generic HMI NLP models to extend their skill in adapting to new HMI sub-system indication patterns (languages $L_{1}\|L_{2}\left\|\ldots \right\|L_{n}$) thus, avoiding learning from scratch (shorter training time).

(8) $$\begin{array}{c} L_{1}\left\|\ldots \right\|L_{n}=\left\{s:s\;\epsilon \;\sum^{*}:s↑i=s_{i}\;\epsilon \;L_{i},i=1,\ldots ,n\right\} \end{array}$$

In summary, RW framework for a HMI DES allows the application of NLP algorithms by specifying a generator automaton that can specify a DES by control language. The generator language dictionary is a finite event set containing alphabets or words equivalent to the finite range of discrete unique values various HMI features can take on. Patterns or sequences of events make the sentences or states of the HMI DES language and cause HMI to transition from one state to another. NLP algorithm are used to create deep learning models to estimate state mapping mechanisms (δ) through supervised training. Once trained, such a model can translate multi-length sentences from one controller language into another.

## 4. NLP Model Design

This section discusses two model designs under NLP architecture that have been evaluated below. Model input, output variable notations and parameter nomenclature are listed in Table 1 for quick referencing.

### 4.1. Seq2Seq—LSTM Encoder-Decoder

Sequence-to-Sequence (seq2seq) represents a class of machine learning problems that entail model training to generate fixed length output sequence of symbols when given fixed length input sequence of symbols. There are no restrictions placed on any particular length of either input or output sequences. An *Encoder-Decoder(EncDec)* architecture is adept for seq2seq class of problems, for example, NLP (machine translation), image captioning, sentiment analysis, and so forth.

**Figure 7:**
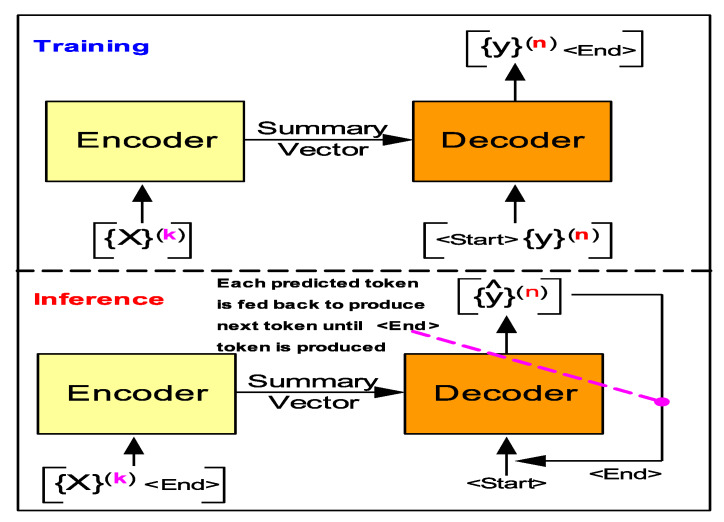
Model Under Training Vs. Inference. Encoder-Decoder Model are trained together using teacher forcing—decoder input is replaced with ground truth tokens which results in exposure bias. During inference previous decoder output is fed forward as input to output next token.

In the general case of machine translation using *Encoder-Decoder(EncDec)* architecture (Figure 7), during the model training mode, entire input sequence of one language ($L_{1}$) is required along with the output sequence of the target language ($L_{2}$). However, during translation (**inference** mode) each previously predicted symbol is required to be fed forward to *decoder* input to produce subsequent symbols until a sequence end token is produced.

**Figure 8:**
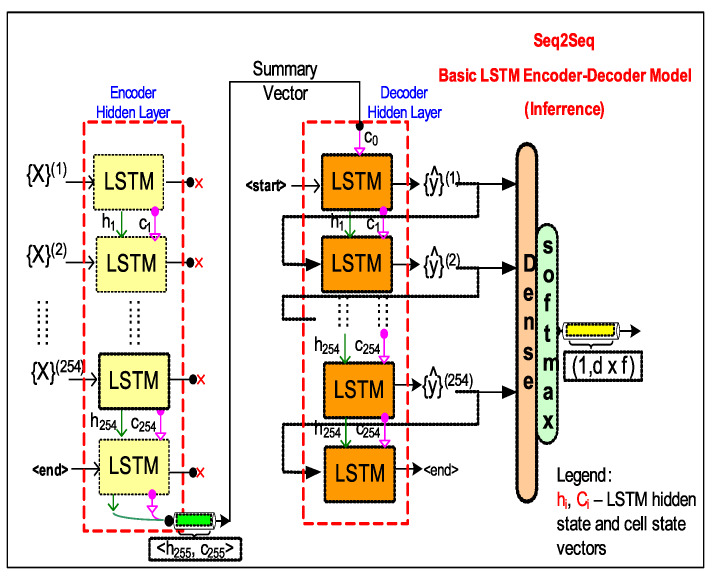
Basic LSTM (RNN) based encoder-decoder model for seq2seq applications. Sequence of input tokens, $X^{\left(K\right)}$ are fed to the encoder LSTM stack, which pass intermediate hidden ($h_{k}$) and cell state ($c_{k}$) vectors in between. The last layer hidden state vector (h,c) is the *summary* vector that captures the essence of entire input sequence which initializes the first decoder LSTM cell including a *<start>* token as input. The output or each LSTM cell is fed back as input to subsequent LSTM cells to generate target tokens (${\hat{y}}^{\left(N\right)}$) one time step at a time until *<end>* token is generated (during inference).

In case of a *LSTM-EncDec* model (Figure 8) the *encoder* LSTM layer functions to produce a fixed size internal representation (summary vector) of the given input string of symbol sequence of any length truncated by a special <End> token (it triggers decoder to start translating). The summary vector is obtained as a result of accumulated hidden states from each LSTM cell in the *encoder* hidden layer as a result of processing the entire input sequence of symbols. Therefore, the final summary vector consists of hidden state and cell output <(h,c)> from the last *encoder* LSTM cell. The LSTM *decoder* uses the summary vector to initialize its first cell state, with the desired effect of incorporating contextual information representing the entire input sequence that aids in predicting the next translated symbols.

#### 4.1.1. LSTM-EncDec—Training Phase

Supervised training of *LSTM-EncDec* is to maximize the probability ($logP({\left\{y\right\}}^{\left(n\right)}|{\left\{X\right\}}^{\left(k\right)}$) (9) of generating the target *n*-ahead samples of target HMI event sequence (${\left\{y\right\}}^{\left(n\right)}$) given the entire previous *k*-lagged samples of input sequence (${\left\{X\right\}}^{\left(k\right)}$) context. This is done by the learning (or optimizing) the RNN parameters (ϕ) of the *decoder* in *LSTM-EncDec* model to maximize the *log* probability of each target HMI event token ($y_{t}$) given a fixed summary vector ($h_{k}$) or the hidden state of final *encoder* cell. Where, $h_{k}$ (11) is a non-linear function of each input event (token) (${\left\{X\right\}}^{\left(k\right)}$) and corresponding previous hidden states ($h_{t-1}$) from other *encoder* RNN/LSTM cells.

The *LSTM-EncDec* model for current HMI DES seq2seq application is trained by using a method referred to as *Teacher forcing* (10) as shown in Figure 7, which is a dynamic supervised training task with input/output sequence pairs being: (${\left\{X\right\}}^{\left(k\right)}$/${\left\{y\right\}}^{\left(n\right)}$) from source and target HMI DES languages ($L_{1}$, $L_{2}$) respectively, to jointly train the *encoder-decoder* system. Where, ${\left\{X\right\}}^{\left(k\right)}$ is framed as a sequence of HMI events from *k* time steps in past and ${\left\{y\right\}}^{\left(n\right)}$ as target HMI sequence of events *n* time steps-ahead in future. Under *Teacher forcing*, a RNN based *seq2seq* models is trained by replacing the previous predicted output of a *decoder* cell $y_{t-1}$ by the actual (ground truth) or *teacher* supplied value as input to subsequent cells for learning. That is, the feed-forward links between *encoder* RNN cells is bypassed by injecting teacher supplied signals. This is analogous a *teacher* who corrects the student at every step of a task sequence; instead of allowing the student to complete the entire task sequence fully and then learn from her mistake.

**Figure 9:**
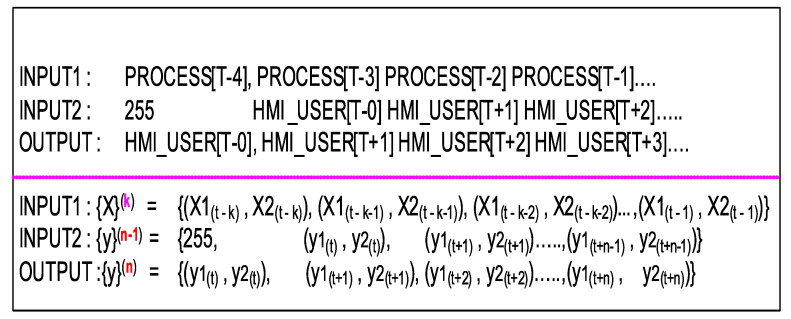
Teacher Forcing—Training Data Stream. INPUT1 is the encoder training input which is fed as the source sequence. Teacher forcing training data stream requires decoder input (INPUT2) and expected ground truth output (OUTPUT) be offset by 1 time-step by inserting a start of sequence (<Start>) marker token (255). Optionally a <End> marker may be inserted in training output sequence (OUTPUT).

Specifically in our design, sequence of one-hot encoded vectors are constructed for *encoder*: INPUT1—${\left\{X\right\}}^{\left(k\right)}$, *decoder*: INPUT2—${\left\{y\right\}}^{\left(n-1\right)}$ (suffixed with a start token) and *decoder*: OUTPUT—${\left\{y\right\}}^{\left(n\right)}$ (as expected target output) as depicted in Figure 9. The sequence of one-hot encoded vector corresponds to a sequence of events (a.k.a forms *sentences* from $L_{1}$ and $L_{2}$ languages) that are HMI DES states. Each one-hot encoded vector has a fixed dimension (255 bits) that is same as the dictionary event size and represents a particular value of the HMI indication feature(s) (X1,X2). The output of *decoder* is the target translated *sentence* in $L_{2}$ that corresponds to the predicted next state of HMI DES, given the previous HMI state presented as INPUT1/INPUT2 to *encoder* and *decoder* respectively. One caveat with INPUT2 is that it is offset by one time step compared to expected OUTPUT sequence owing to inclusion of a *<start>* suffix token, which triggers the *decoder* to begin translation. Note, that the required *<end>* token is not included as it is implicit in our design by having a fixed length input and output sequence(s) (However, this is not a hard restriction as the model is able to support variable length sequences).

(9) $$\begin{array}{c} \begin{array}{c} \mathrm{LSTM}-\mathrm{EncDec}\mathrm{Model}\\ logP\left({\left\{y\right\}}^{\left(n\right)}|{\left\{X\right\}}^{\left(k\right)}\right)=\sum_{t=0}^{N}logP\left(y_{t+1}|y_{t},X;\phi \right), \end{array} \end{array}$$

where:

N=n−1, $y_{0}$ = <start> token,
ϕ—learned parameter.

(10) $$\begin{array}{c} \begin{array}{c} \mathrm{Teacher}\;\mathrm{Forcing}\;\mathrm{Training}\\ logP\left(y_{t+1}|y_{t},X;\phi \right)=logP\left(y_{t+1}|h_{t};\phi \right) \end{array} \end{array}$$

(11) $$\begin{array}{c} \mathrm{Hidden}\;\mathrm{State}\;\mathrm{or}\;\mathrm{Context}\;\mathrm{Vector}\\ h_{t}=\left\{\begin{array}{cc} f(X;\phi ), & \mathrm{if}\;t=0\\ f(h_{t-1},y_{t-1};\phi ), & \mathrm{otherwise} \end{array}\right. \end{array}$$

where:

$h_{t},\phi$—hidden vector, learned parameter respectively.

#### 4.1.2. LSTM-EncDec—Inference Phase

The *LSTM-EncDec* model once trained using *Teacher forcing* may be used to infer (Figure 7) the expected HMI event(s) of the target HMI state (translated language pattern) ${\hat{y}}^{n}$ one step at a time. Translation begins once the <start> is fed to the first *decoder* cell whose internal cell state has been initialized by the summary vector from the *encoder*. Subsequent, target event tokens are inferred by feeding as input the partial target HMI event pattern that is built by appending previously predicted event tokens to it. The downside of this is if any previous inferred token is incorrect, subsequent predictions will be off.

Moreover, the basic *LSTM-EncDec* model has also shown to be limited [42] in its translation skill for longer length sequences, owing to a bottleneck associated with the final fixed-length summary vector that only serves as a coarser context of the source pattern for the *decoder*. To overcome this problem, *Attention* mechanism was introduced.

#### 4.1.3. RNN Attention Mechanism

The encoder-decoder seq2seq RNN based models can be made more versatile in doing machine translation of longer source patterns by using *Attention* mechanism. Attention in general was proposed to provide more feature-full encoding of the source HMI event pattern from which finer grained context vectors can be obtained. This allows the *decoder* to apply varying degree of *Attention* to every input event token with its corresponding *encoder* hidden states—referred to as *Keys*(*K*) or *Values*(*V*), in the source sequence for predicting each HMI event token in target sequence, given previous decoder hidden state—referred to as *Query*(*Q*).

(12) $$\begin{array}{c} \begin{array}{cc} \mathrm{Attention}\;\mathrm{Mechanism}\\ e_{t,i}=f\left(Q_{t},K_{i}\right) & \;\mathrm{Similarity}\;\mathrm{Score},\\ a_{i}=softmax\left(e_{t,i}\right) & \;\mathrm{Attn}.\;\mathrm{Weight}\;\mathrm{Vector},\\ Attention\left(Q_{t},K,V\right)=c_{t}=\sum_{i}a_{i}V_{i} & \;\mathrm{Attn}.\;\mathrm{Context}\;\mathrm{Vector}. \end{array} \end{array}$$

**Figure 10:**
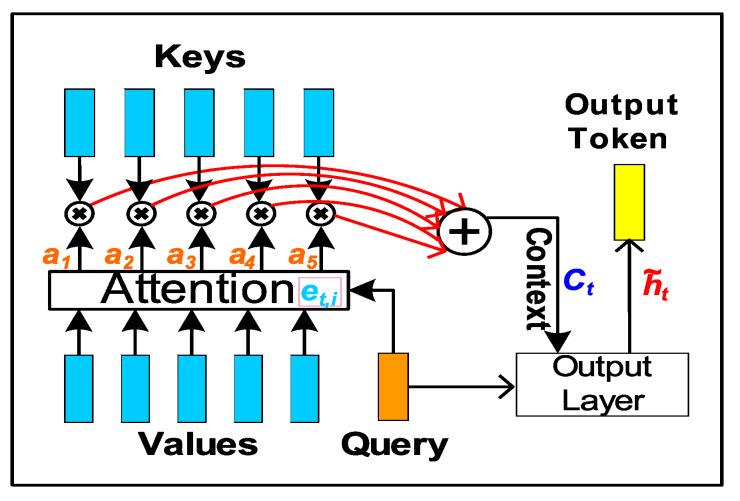
Attention Mechanism (conceptual). Allows the decoder to learn how much influence each **Key-Value** (input tokens) has on a given **Query** (target token) dependence which allows accurately predicting next output token. (Note: all subscripts span length of input sequence 1..N. Above example is just for a specific case where N = 5).

The *Attention* mechanism [26,29] (Figure 10) results in constructing a attention context vector, $\mathrm{Attention}\left(Q,K,V\right)=c_{t}$ (12), as a sum of encoder hidden states weighted with normalized attention weights ($softmax\left(f\left(Q,K_{i}\right)\right)=\alpha_{ti}$) used for predicting each output HMI event token. It starts with an alignment model ($f(Q,K_{i}$) to learn a similarity score ($e_{ti}$) between all hidden states ($h_{i}$) of the *encoder* at time-steps i=1,…,k of the source sequence with respect to the decoder’s output hidden state $s_{t-1}$, at previous time step t−1. The alignment score $e_{ti}$ essentially captures how relevant (or similar) each encoded state $h_{i}$ ($h_{1}$, $h_{2}$, …, $h_{k}$) (*Key* and *Value*) is to the decoder hidden state from previous time step ($s_{t-1}$) (*Query*), which can be used to infer decoder output at next time step (T=t). Each score $e_{ti}$ is then normalized using *softmax* function in order to be used as probability value to indicate how likely each encoded input HMI token is relevant to the current decoded output HMI event token. The normalized scores are also referred to as attention weights ($\alpha_{ti}$). The alignment model is implemented as a feed forward single layer perceptron that is jointly trained ($W_{a},U_{a},v_{a}$ trainable matrices) Equation (13) with the rest of the translation encoder-decoder seq2seq model to learn various attention weights for every encoded tokens with respect to an output token. The context vector ($c_{t}$) is calculated for every decoder output step (t), it represents the sum effect of all encoder hidden states (i=1,…,k) weighted with *expected* attention weights ($\alpha_{ti}$)—this captures the relative influence of each HMI event token in input sequence $X^{\left(k\right)}$ on an output HMI event token in target sequence (${\hat{y}}^{\left(N\right)}$). The above attention mechanism is based on global attention mechanism using additive (feed-forward) similarity function as initially proposed by Reference [29] and described in Equation (13).

(13) $$\begin{array}{c} \mathrm{RNN}\;\mathrm{Attention}\;\mathrm{Mechanism}\\ \mathrm{Similarity}\mathrm{Score}:\\ e_{ti}=a\left(s_{t-1},h_{i}\right),\\ \mathrm{Attention}\mathrm{Weight}:\\ \alpha_{ti}=\frac{exp(e_{ti})}{\sum_{i=1}^{K}exp\left(e_{ti}\right)},\\ \mathrm{Additive}\mathrm{Alignment}\mathrm{Model}:\\ a\left(s,h\right)=v_{a}^{T}tanh\left(W_{a}s_{t}+U_{a}h_{i}\right),\\ \mathrm{Context}\mathrm{Vector}:\\ c_{t}=\sum_{i=1}^{K}\alpha_{ti}h_{i}, \end{array}$$

where:

$W_{a},U_{a},v_{a}$—trainable input, hidden and output layer weight matrices respectively.

### 4.2. Seq2Seq—CNN Encoder-Decoder

Recent trend in departure [43,44] from using RNN based encoder-decoder (e.g., *LSTM-EncDec*) models for seq2seq prediction to the approach that combines *Attention* with convolutional neural networks (CNN), has shown to outperform RNN based models in area of machine translation. RNN based models are sequential in nature and generally require more memory bandwidth resources than computational units. In contrast CNN *Attention* models offers themselves as better suited for parallel and in-memory processing computational architectures [45].

The proposed model, here referred to as *Trident*, draws inspiration from the design recipe “Embed, encode, attend, predict” by [46] in 2016, for building NLP CNN based models. It also utilizes design concepts from transformer [26] and residual network (ResNet) [47] models to build a scalable network that provides comparable or better performance than sequential *LSTM-EncDec* model with single-headed attention layers evaluated herein.

#### 4.2.1. Trident—Encoder

**Figure 11:**
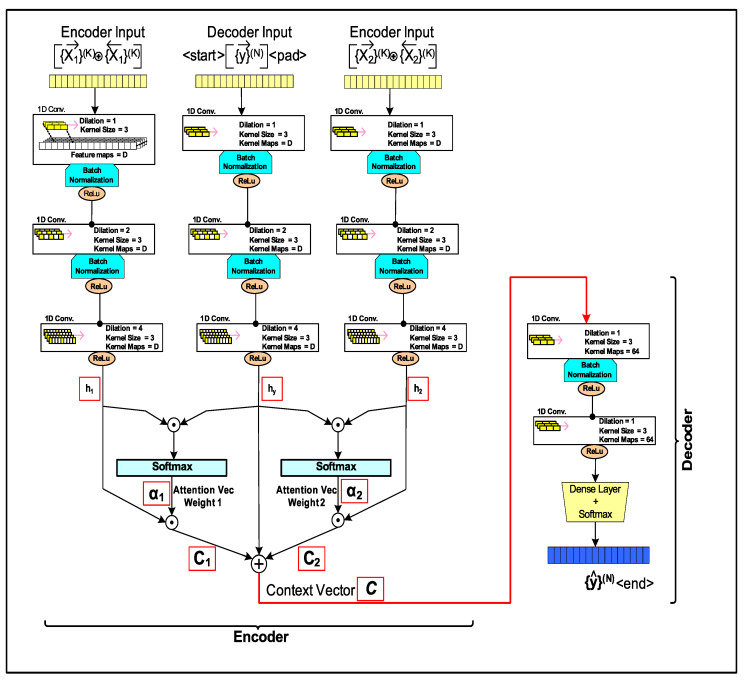
Trident Model: Contains stacked 1D convolutional neural network (CNN) layers to build a parallel architecture that uses dot product attention layers for its encoder-decoder network. Intermediate deeper CNN layer have larger (1,2,4) dilated kernel filters to increase the receptive filed of the convolution maps to capture longer dependencies.

Supervised training of the *Trident* (Figure 11) requires the same number of inputs (encoder: INPUT1, decoder: INPUT2) and target output (OUTPUT) as required by seq2seq *LSTM-EncDec* model, discussed previously. However, the encoder inputs (INPUT1) is framed slightly different, where each HMI indication feature sequences of ${\left\{X\right\}}^{\left(k\right)}$ (X1, X2) are made bi-directional by concatenating the corresponding sequence in reverse order (e.g., ${\vec{\left\{X_{1}\right\}}}^{\left(k\right)}$ concatenated with ${\overset{\leftarrow }{\left\{X_{1}\right\}}}^{\left(k\right)}$ ). This doubles the length of the resulting training input vector of each HMI indication feature vector.

In addition the target HMI event sequence (${\left\{y\right\}}^{\left(n\right)}$) is introduced as input to the *tridentdecoder* in a *progressive* way, during training, that is one new target token every few epochs (each target token from the training set is appended to intermediate training sets). The overall ${\left\{y\right\}}^{\left(n\right)}$ target sequence is padded to maintain a uniform sequence length. Therefore, the total number of training epochs is equally split between all (*n*) tokens of the target HMI event sequence (${\left\{y\right\}}^{\left(n\right)}$). Progressive sequence training is done to allow the model to train in a way, mimicking its functionality during translation or performing inferences. This technique is to overcome exposure bias [48] (training-inference discrepancy) in training a non-sequential CNN based model using *Teacher forcing*. Results show improvement while predicting the output sequence one token at a time during the inference phase, as the model *encoder* over-fitting is reduced. The model is also more resilient to begin translation using a partial sequence consisting of only a few previously output tokens generated by the *decoder*. A *<start>* token is appended to each *decoder* input and an *<end>* token to each target HMI event sequence similar to training the *LSTM-EncDec* models. Again, the rationale for these tokens is to offset (lead) the *decoder* input sequence during training by one-time step (t−1) compared to the target HMI indication sequence.

#### 4.2.2. Trident—CNN Layers

The proposed *trident* model design, as depicted in Figure 11, includes a parallel pathway consisting of several convolutional neural network (CNN) layers to extract features from each input and the target HMI indication sequences. A 1D-convolution (CNN) layer is utilized since it is more conducive for processing sequence-based data sets, rather than images that have spacial information in two-dimensional space. The size of the convolution kernel (filter) is has been chosen (Kernelsize=3) to be a value that is commonly used most CNN based models for language translation.

Number of feature (filter) maps generated in encoder for each convolution layer is set to the dictionary size (Featuremaps=D) of the HMI model input and output indication languages ($L_{1}$, $L_{2}$) including the special *<end>* and *<start>* tokens. Therefore, *D* feature map vectors are generated, followed by the application of a non-linear activation function (rectified linear unit (ReLU) is used currently). In order to minimize the model, over-fitting regularization is required. Regularization in CNN can be achieved by *dropout* connection, which randomly drops connections between convolution layers, implemented in the CNN dense (fully connected) final output layer in order to avoid saturation of gradient during backpropagation. Nevertheless, *dropout* does lead to “un-learning” or decimating the previously learned weights, which is an issue in case the training data set is small. Instead, *batch normalization* [49] was used between each convolution layer as a way to regularize each convolution input pathway of *trident* model while it yields other benefits such as reduction of covariate shift by normalizing the activations of each layer and speed up learning of CNNs.

**Figure 12:**
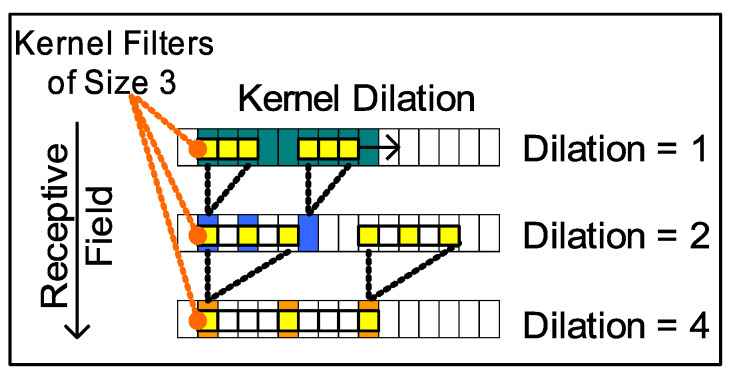
Dilation of kernel filters increases the receptive field of the higher subsequent convolution maps.

Unlike the RNN *encoder-decoder* (*LSTM-EncDec*) models, basic CNN encoders are not sequential and do not learn sequence dependencies in long patterns by default as efficiently as LSTM layers can. This limitation can be overcome by arranging the longer input sequences in spatially close proximity such that the resulting convolution feature maps may capture sequential patterns and related dependencies. In order to avail this, each input sequence has been made bi-directional by concatenating the previous sequence in reverse order (as stated previously). Another design feature is to use dilated kernel filters in intermediate convolution layers, which further allows the Trident to increase its *receptive* field, as depicted in Figure 12, to capture longer sequence dependencies.

#### 4.2.3. Trident—Attention Layer

Attention mechanism allows the CNN decoder to use various sub-regions of the input to draw the output token. In the proposed model CNN *Trident* model (Figure 11), firstly the similarity of the CNN layer internal representation vectors ($h_{1}$, $h_{2}$) for bi-directional *encoder* input ${\left\{X\right\}}^{\left(k\right)}$ sequence(s), against the target *decoder* input sequence (${\left\{y\right\}}^{\left(n\right)}$) internal representation ($h_{y}$) is obtained by performing vector dot products. The result of dot product generates similarity scores for each sub-segment of the input HMI event sequence against the target HMI event sequence. The following *softmax* activation is used to normalize the similarity scores to a probability distribution over all segments of the input sequence, which can be used as the attention weight vector ($\alpha_{1}$,$\alpha_{2}$). Secondly, the attention weight vectors are combined with the *encoder* input ${\left\{X\right\}}^{\left(k\right)}$ CNN layer internal representation vectors ($h_{1}$, $h_{2}$), which ultimately applies the attention (focus) on various segments of the HMI sequence ${\left\{X\right\}}^{\left(k\right)}$ given the target HMI sequence ${\left\{y\right\}}^{\left(n\right)}$. Lastly, all context vectors ($C_{1}$,$C_{2}$) are concatenated along with the internally represented *decoder* input $h_{y}$ to obtain the final context vector C.

#### 4.2.4. Trident—Decoder

Training of the *Trident decoder* is done along with the *encoder* using progressive target sequence as per *Teacher forcing* technique. The *decoder* learns to use the context vector (C) to output the next token (t=T) of the target sequence, given the partial sequence containing target tokens t=0 to t=T−1 (where, *T* is the sequence index of token in target sequence of length T=N) as the input to the *decoder*. The context vector C captures the *encoderattention* or influence of the input sequences (${\left\{X\right\}}^{\left(k\right)}$) on each partial target sequence ${\left\{y\right\}}^{\left(t-1\right)}$, which are introduced to the *decoder* progressively during training beginning with a <start> token marker. The ground truth target sequence is also required and is same as the *decoder* input sequence but is offset by one time step ahead ending with <end> token.

Inference is carried out similar to model training, where various HMI indication parameter sequences of length *K* (${\left\{X\right\}}^{\left(K\right)}$) are provided as input to the *encoder* as bi-directional sequences. The *decoder* input initially starts off with just the <start> token padded to the target HMI sequence of length (*N*). Subsequently, as the *decoder* outputs a new token (${\left\{\hat{y}\right\}}^{\left(n-1\right)}$), it is appended to the sequence which is fed back as input to the *decoder*. The context vector (C) evolves with the previously output target token and is used to generate a new target token (${\left\{\hat{y}\right\}}^{\left(n\right)}$) by the *decoder* until the <end> marker is output.

*Trident* decoder is implemented with two back to back 1D CNN layers with the first layer having batch normalization. Both CNN layers have a fixed number of kernel filters (64). A dense layer with *softmax* activation is used to reshape to a target sequence of length *N* tokens, which are one-hot encoded to dictionary size D=255 (including sequence de-marker tokens).

### 4.3. Curriculum Training

*Curriculum* training [50] overcomes the limitations of *Teacher* forcing and makes the model more versatile (generalized) during training and accurate during inference. Training the seq2seq models discussed above using *Teacher* forcing, inherently prevents the models from being generalized for the inference phase. The issue is referred to as *exposure* bias (training-inference discrepancy [48]), where the models are trained using actual ground truth target tokens (${\left\{y\right\}}^{\left(n\right)}$) each time step, while during inference the model *decoder* is fed back previously predicted token (${\left\{\hat{y}\right\}}^{\left(n\right)}$). Thus, limiting the skill of the seq2seq models to predict target tokens during inference over longer forecast windows correctly. As, once an incorrect token is output, it may throw all prediction of subsequent target token sequence off completely.

*Curriculum* training [50] entails seq2seq model *decoder* to be trained in mini-batches where both actual ground truth and predicted tokens ({y}, $\left\{\hat{y}\right\}$ receptively) are utilized. A sampling schedule is followed to systematically control the probability (per epoch) in training mini-batch of how many of either target or formerly predicted tokens are to be fed to the decoder during training. Namely, at each training epoch based on the selected probability schedule (linear, exponential or inverse sigmoid decay) [50] either (actual) ${\left\{y\right\}}^{\left(n-1\right)}$ or last predicted ${\left\{\hat{y}\right\}}^{\left(n-1\right)}$ is used. The sampling schedule is set up to decay the probability ($\epsilon_{i}$) of selecting ground truth ({y}), vs. previous predicted token ($\left\{\hat{y}\right\}$) decreases as training epoch count increase in a mini-batch (e.g., exponential $\epsilon_{i}=k^{i}$ decay, where k<1 and *i* is epoch index). This allows the model to get trained by *teacher* forcing in the beginning and slowly transition to using predicted target token output from the trained *decoder* trained thus far, hence improve generalization and reduce over-fitting.

*Trident* is evaluated with using both *teacher* forcing and *curriculum* learning with linear decay sampling schedule. It is noteworthy to state here both training techniques are used in conjunction with the progressive sequence training, which rather controls how the *decoder* input is built progressively as described previously.

## 5. Experiments

### 5.1. Data Generation

**Figure 13:**
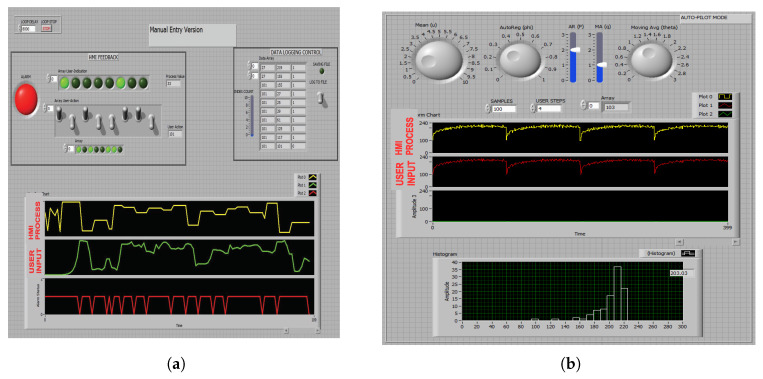
(**a**) *ht*-MI application in Manual mode: Process values are generated using a first-order AR(α), MA(β) process with Gaussian noise ($\epsilon_{t}$), periodic trend and seasonal components Equation (14). Manual mode: Process values are displayed via array of lamp indicators. The user tracks indication patterns by manually setting toggle switches in same pattern until alarm indication (red lamp) goes off while user actions are logged each time step. (**b**) *ht*-MI application in Auto Pilot mode: In Auto-Pilot mode, user tracking response to process values is modelled using a PI controller with random proportional gain. Human Machine Interface (HMI) process and user response is captured as time series data.

For the scope of this experiment a hypothetical HMI (*ht*-MI) application (Figure 13a,b) was built using *National Instruments Labview 8.0* standard function blocks. The *ht*-MI displays values of an arbitrary simulated process as a pattern of 8 indication lamp states (*8-bit*), which the HMI user is required to visually track and manually set an array of 8 rocker style toggle switches either ON or OFF corresponding to each indicator lamp state being either ON or OFF. The process state evolves as per synthetic data sample generator Equation (14)—which is a first-order linear autoregressive (AR or α), moving average (MA or β) process, with Gaussian random noise ($\epsilon_{t}$) and an adjustable period of sinusoidal seasonal component. In addition, modulus of natural logarithm is added to introduce an auto resetting trend component to TS. The parameters of the sample generator Equation (14) are as listed below in Equation (15).

Synthetic data generated by the *ht*-MI application is based on the weakly stationary time-series assumption made for HMI state space, as discussed previously in Section 3.2. Moreover, the intent of this experimental setup is to be representative of the complexity and volume of data obtained from real HMI systems and operator actions at a fundamental level, in order to demonstrate the principle of modeling HMI state sequences via NLP models and exploring its potential ability to scale-up in future.

(14) $$\begin{array}{c} \begin{array}{c} \mathrm{Synthetic}\;\mathrm{Data}\;\mathrm{Model}\\ Y_{i}=\mu +\alpha Y_{i-1}+\beta \epsilon_{t-1}+\epsilon_{t}+\sin\frac{2\pi t}{\kappa }+ln\left[\left(t-\kappa \right)\left\lfloor \frac{t}{\kappa }\right\rfloor \right] \end{array} \end{array}$$

where:

μ,α,β,κ—Mean, Auto-regression, Moving-Average, seasonal trend resetting parameter,
$\epsilon_{t}$—Gaussian noise term.

(15) $$\begin{array}{c} \begin{array}{c} \mathrm{Process}\;\mathrm{Parameters}\\ \mathrm{Gaussian}\;\mathrm{random}\;\mathrm{noise}\left(\epsilon_{t}\right):\left(\mathrm{seed}=-1,\mu =0,\sigma =0.5\right)\\ \mathrm{Seasonal}\;\mathrm{component}\;\mathrm{period}\left[\frac{t}{\kappa }\right]=\frac{\mathrm{time}-\mathrm{step}}{200} \end{array} \end{array}$$

**Figure 14:**
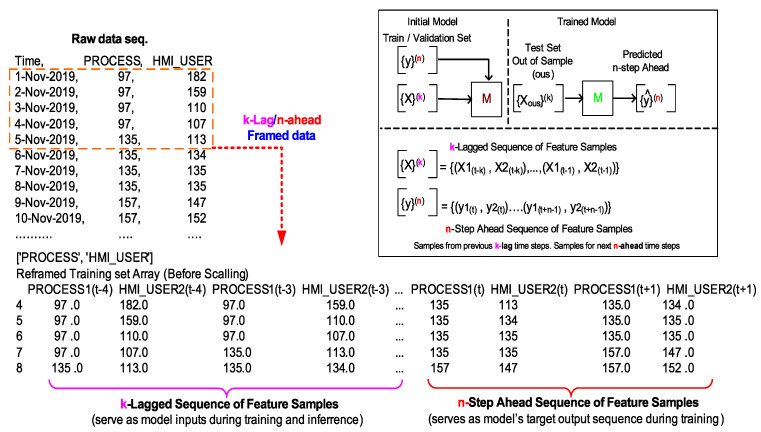
Snippet of raw time-series data set generated from *ht*-MI application. *PROCESS* and *HMI_USER* values are restricted to 8-bit integers (arbitrary time stamps generated). The raw data series are framed into supervisory training data as desired for source (*k-lag*) and target (*n-ahead*) length of sequence pairs.

This setup allows for generating two types of raw data sets under: manual entry and auto-pilot modes. In the manual entry mode (Figure 13a) HMI user manually set switch states are captured against the process indicator (lamp) values. Each time step sample (row) consists of a 2-tuple vector as shown in Figure 14. In the auto-pilot mode (Figure 13b) the user response is modeled using a PI (proportional-integral) controller with its proportional gain set each time step randomly within a fixed range, that was determined by trail-error to closely match the author response rate, observed in manual entry mode. Each state value is restricted to *8-bit* in manual entry mode in auto-pilot mode.

### 5.2. Raw Data Sets

The raw HMI data sets are generated from either two sources: manual and auto-pilot modes. Each data set (Figure 14) contains approximately (adjusted as desired) 4K samples with columns: *Time*, *PROCESS*, *HMI_USER*. Time index is currently stored as date strings while the data columns hold time sample tuples.

**Figure 15:**
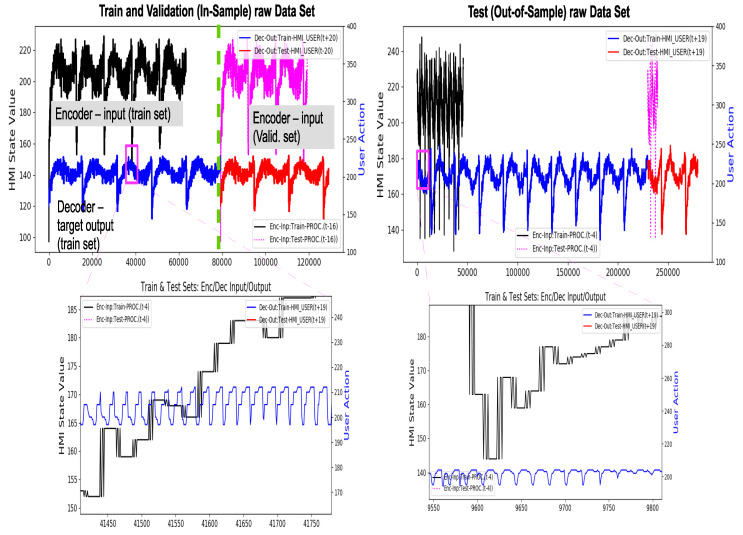
Raw Training/Validation and Test data sets for doing In-Sample and Out-of-Sample model performance evaluation, respectively. Below each plot are zoomed-in sections of the over-all sequence patterns to show the underlying patterns. Test data set uses a slightly slower HMI operator response: The PI controller’s integral constant is changed to 6 steps compared to 4 steps used for original training set).

For this experiment, two (training and test) HMI raw data sets were generated separately using auto-pilot mode with slightly different auto-regressive moving average (ARMA) time series (α, β, μ) parameters listed in Equation (16) for the process generator Equation (14) along with random proportional gain parameter for the (PI) operator response generator. The resulting two raw data sets were framed (arranged) into (*k* lag and *n-step* ahead) supervised training and test time series sample data sets with the effective series mean remaining in range 198 to 255 as shown in Figure 15. The rationale for keeping ARMA process generator is to ensure the synthetic data set do not fall outside the fixed vocabulary size (HMI states: 0 to 255) pre-selected for this experiment. The test data set contains similar HMI process patterns but slightly different HMI user patterns which is obtained by changing the PI integral control reset constant from 4 to 6 steps, effectively slowing down the operator response rate. This is conducive for evaluating out of sequence prediction performance of the trained models.

(16) $$\begin{array}{c} \begin{array}{c} \mathrm{HMI}\;\mathrm{Synthetic}\;\mathrm{data}\;\mathrm{Model}\;\mathrm{AR}-\mathrm{MA}\;\mathrm{params}.\\ \mathrm{Training}/\mathrm{Test}\;\mathrm{Set}:(\alpha ,\beta ,\mu )=(0.6,-0.1,4),\mathrm{mean}\;=\;198\\ \mathrm{Traing}/\mathrm{Test}:\left(\mathrm{PI}\;\mathrm{reset}\;\mathrm{constant}\right)=4,6\;\mathrm{Steps} \end{array} \end{array}$$

### 5.3. Supervised Learning—Data Framing

Supervised learning often requires the raw data sets to be re-framed as input and target output data sets that are representative of the underlying process to be modeled. For this experiment the raw time-series HMI state feature data is framed as a short sequence of values arranged from previous *k* time steps (Figure 14)—referred here with notation ${\left\{X\right\}}^{k}$ as the training sequence (containing both process $X_{1}$ and user input $X_{2}$ features). This lagged sequence of patterns serves as the training, validation, and test data sets for the required prediction model.

Similarly, the ground truth or target model output is framed as a short sequence of values taken from next *n-step* ahead times—referred to here with notation ${\left\{y\right\}}^{n}$ (containing both process and user input features). The *n-step* ahead sequence of patterns also needs to accompany the training and validation sets as expected output patterns for the required supervised training.

Finally, trained prediction model shall be able to predict *n-step* ahead samples of HMI state feature vector (Figure 14)—referred to here with notation ${\left\{\hat{y}\right\}}^{n}$, when *k*-lagged samples (${\left\{X\right\}}^{k}$) are provided as input.

### 5.4. Baseline Model—Persistence Score

**Figure 16:**
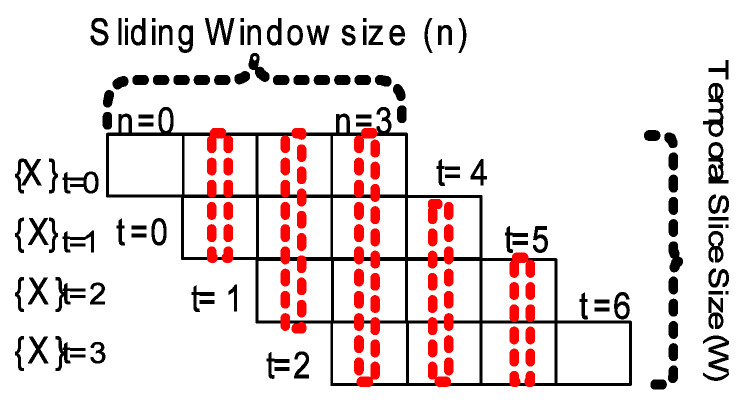
Rolling Window Forecast for n-ahead (e.g., n = 3) Forecast for each input sequence ${\left\{X\right\}}_{t}$. Sub-samples from previous *W* predicted sequences at each temporal (red vertical stripes) slice in sliding windows can be combined appropriately (e.g., averaged, max or min polled, etc.) together for calculating rolling window forecast errors.

Persistence model (also referred to as naive or walk-forward forecast) is used to ascertain the baseline forecast error estimate for a given time series data. Persistence algorithm shifts (lags) a given time-series data by *p* steps in time, and uses it as the input to an ideal prediction model (Figure 16). An ideal prediction model outputs the original data series exactly *p* steps out of phase. Root mean square error is a common metric used to measure the standard deviation of the residuals between predicted and expected samples. In the case of persistence forecast, root-mean-square (RMSEp) error is dependent on the correlation between the lagged expected samples.

For example, when p=0, the RMSEp=0 is as expected, that is, the persistence model will output the next step sample value in sync with the expected output when no shift or lag. *RMSEp* value is generally used as an upper bound for the forecast error or selection criteria for any candidate forecast model to be considered skillful, that is, a selected, trained model must yield a lower forecast error (RMSE<RMSEp) than the persistence error for same *n-step* ahead prediction.

(17) $$\begin{array}{c} \mathrm{Rolling}\;\mathrm{Window}\;\mathrm{RMSE}\;\mathrm{Metric}\\ \begin{array}{c} RMSErw=\sqrt{\frac{\sum_{i=1}^{N}{\left(y_{i}-\frac{1}{W}\sum_{k=0}^{W}{\hat{y}}_{k}\right)}^{2}}{N}} \end{array} \end{array}$$

where:

W,n,N—temporal slice size, sliding window size, total number of samples respectively.

Additionally, a rolling window (rollWin) root mean square error RMSErw such as *rollWin.RMSEp* for persistence model can be calculated using Equation (17). This metric combines samples from previous *W* predicted sequences of *n-step* ahead sample window size. Sub-sequence samples that fall in the same temporal slice of length *W* (Figure 16) as the *n-step* window advances one time step ahead are are summed and scaled by *W* size (generally W=n is assumed). For instance, with ${\left\{X\right\}}_{t}=2$ as input to model, the predicted sequence will yield *4-step* ahead samples for t=2 to t=5 when n-step=4. For each temporal slice (e.g., t=2 to t=5), the predicted sub-sample values are summed vertically and scaled by W=4, these rolling window sub-sample values are then used to calculate the root mean square error against each expected $y_{i}$ (i=0..N) samples. Rolling or sliding window forecast error (RMSErw) is lower, such as seen in case of *rollWin.RMSEp* persistence score (Table 2), owing to the beginning and ending trailing temporal slices having less than *W* number of sub-samples.

The main intuition behind using a rolling window metric is to proportionally combine the errors in redundant predicted sub-samples resulting from generating n−step ahead predicted samples $\hat{y}$, while advancing the prediction window one-time step at a time.

**Figure 17:**
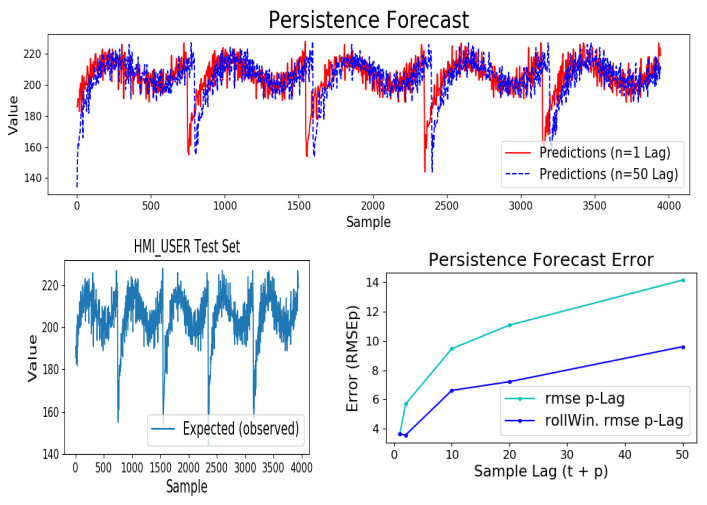
Persistence (RMSEp) score for a training/test data set for *p*-lag Persistence model forecast samples.

RMSEp scores for lags (t + 2; t + 10; t + 20; t + 50) are as shown in Table 2. Figure 17 compares both t + 1 and t + 50 lag persistence forecast and also depicts both RMSEp and rolling window RMSEp as it varies with *p-lag* over the test data set that has been used.

### 5.5. Models and Test Cases

Two models are evaluated in this paper. First is a *seq2seq* long short erm memory (LSTM) based *encoder-decoder* model (referred to here as *LSTM E-D*) with a custom attention layer based on additive attention [29] mechanism as described previously in Section 4.1.3. The second model is the 1D CNN based custom model *encoder-decoder* design (referred to here as *Trident*) that uses dot product style attention mechanism as described previously in Section 4.2.3.

The test cases include evaluating performance of above models under two training styles: (1) *Teacher forcing* (2) *Curriculum* (scheduled sample) Training. Teacher forcing for *LSTM E-D* model was previously described in Section 4.1.1 while teacher forcing, used for the *Trident* model was slightly modified, in that it incorporates *progressive* sample injection to overcome the non-sequential nature of the CNN networks as described previously in Section 4.2.1. Curriculum training was previously described in Section 4.3.

Test Cases:

1. *LSTM E-D* Teacher Forcing (TF)
2. *LSTM E-D* Curriculum Learning (CL)
3. *Trident* Teacher Forcing (TF)
4. *Trident* Curriculum Learning (CL)

Each above four test cases were swept over K-Lag=[4,8,16,32] and n-step=[1,2,4,10,20] parameters to train and evaluate the models using raw data set containing 4 K samples with train vs. validation split of 60–30% for the resulting framed dataset (as described in above Section 5.3). The performance values obtained from the train data set is called the *in-sample*(InS) result test samples were selected from the original training set. Another data set (Section 5.2) containing 11 K samples were generated for only re-evaluating the above-trained models under the above test cases independently—which is the out-of-sample (OuS) performance since all models have never seen these test samples during training.

In this application, both InS and OuS model performance are relevant owing to two benefits (1) a lower validation accuracy compared to training accuracy on InS dataset, helps identify model over-fitting (2) A high prediction accuracy on InS dataset may be desired in certain applications where the process HMI states and operator actions are not expected to vary much with respect to each other. This is particularly the case in safety-critical applications. Lastly, model performance (accuracy) using OuS obviously shows the model’s generalization to the test data set.

All models have been custom-built using Python 3.6.7 Keras 2.2.4 API libraries with open source TensorFlow 1.13.1 as its back end implementation.

## 6. Results

### 6.1. Time-Series Metrics

The objective is to accurately model the supervised training dataset sequence of samples. Each sample includes K−Lag past tokens (source language) to produce N−Step ahead tokens (target language) of HMI state (comprising of both process and operator response) feature parameters. Initially, this modeling was treated as a regression time-series prediction problem, therefore rolling window Root-Mean-Square Error (RMSErw) had been used as a metric to measure relative prediction accuracy. Persistence rolling window RMSEp (baseline) scores for the desired N−step ahead predictions (Section 5.4) is used to compare the relative prediction performance of various models.

All *LSTM E-D* models yield better (lower RMSErw) than baseline (RMSEp) persistence score compared to the *Trident* CNN model. In general *LSTM E-D* yields ≈26% higher forecast accuracy than the custom *Trident* model.

Lower RMSErw values are demonstrated for longer K−Lag source sequences. Since, that provides the encoder-decoder more history per sequence to base its prediction on and improve its forecast skill, than avilable for shorter source sequences K−Lag values. Results in Table 3 show *LSTM E-D* generally yields lower RMSErw (e.g., *LSTM E-D*: RMSErw=0 while *Trident*: RMSErw=90.3 for (K-Lag,N-step)=(32,20)) compared to *Trident* model. This is owing to the non-sequential nature of CNN to learn temporal sequences, which is seems to depend on *Trident* depth (number of CNN layers) and combination of source/target sequence lengths. The custom *Trident* CNN model (Figure 11) currenlty utlizes two 1D CNN layers. Nevertheless, current state-of-the-art research reveals CNNs may overcome the limitation by using more deeper models, as such demonstrated by the Transformer model [26].

Time-series Prediction Summary:

- *LSTM E-D* yields consistent lower rolling window RMSE (RMSErw) values than baseline score RMSEp (Table 3) for all InS data test cases (which is desirable performance). While, *Trident* only yields lower rolling window RMSE (RMSErw) values than baseline (RMSEp) for shorter target sequence (N-Step<20) cases, as long as source target sequence is K-Lag<16. A behaviour that is directly related to keeping kernel filter size constant for various source/target lengths.
- *LSTM E-D* generally yields lower RMSErw than *Trident* model. *Trident* models needs to be tuned to work with a particular combination of K−Lag/N−Step source/target sequence lengths.
- *LSTM E-D* and *Trident* model yield comparable Accuracy(tolerance) values for all test cases. Therefore, both models are adept are modeling the general trend of expected future patterns (within a tolerance).
- *LSTM E-D* shows lower (≈53%) validation accuracy than *Trident*. Suggests *Trident* achieves less over-fitting with the same amount of available training data.
- Out-of-sample RMSErw scores are higher than RMSEp (Table 4) for all models, but show consistent trends of values for same K−Lag/N−Step InS test cases. Therefore, InS performance is a good indicator for estimating the performance for OuS datasets.

### 6.2. Prediction Accuracy

In addition, the proposed approach is to transform the time-series modeling as a NLP machine translation problem, which falls under the multi-classification domain of machine learning. Hence, label prediction accuracy measures and bilingual evaluation understudy (BLEU) (translation quality) score have been utilized here.

For the accuracy, *Accuracy(hard)* metric looks for a one-to-one match, each time step, between the class labels of expected and predicted output sequences. Secondly, *Accuracy(tolerance)* is a custom metric that is a relaxed accuracy measure that too makes one-to-one class label comparison, but as long as class labels only do not vary more than a specified tolerance threshold (tol.=0.01%) they are accepted, otherwise rejected as not matching.

Results (Table 3), show the advantage of Curriculum learning vs. Teacher forcing for both *LSTM E-D* and *Trident* models, as the models acquire prediction skills faster (requiring less epochs). Example, for *LSTM E-D* under teacher forcing Accuracy(hard)=100% for (32,20) is reached at Epoch=148, while Accuracy=65.65% is reached at Epoch=81. Similarly, for *Trident* model Accuracy(hard)=28.79% for (32,20) at Epoch=421, while Accuracy=65.22% is reached at Epoch=81.

BLEU *1-gram* to *4-gram* overlap scores were calculated to ascertain each model’s language translation performance using previous *K-Lag* samples of HMI process (source language) to N−ahead samples of HMI operator action sequence (target language). BLEU algorithm requires two sets of reference and candidate sentence corpus. In this case, the reference sentence set contains the expected N−ahead samples of HMI operator sequence, and the candidate set contains the actual predicted N−ahead samples of HMI operator sentence.

In Table 3, BLEU scores have been averaged over all sequences in the evaluation data set for each listed test case. The perfect BLEU score is a value 1. We BLEU-1 as an individual 1-gram score to simply to account for the presence of all expected tokens in candidate sentences and that the expected sequence length matches the reference sequences. In-sample(Table 3) results indicate all models show a higher (>0.95) BLEU-1 score for all test cases, indicating all models are skillful enough to generate the expected sequence length with all required tokens (irrespective order).

Similar to BLEU-1, BLEU-2 individual score looks at 2-gram (token) overlap (applicable for N−Step >1 ahead sequences), that is, for expected token pairs must appear in the same pair order as in reference sequences. For in-sample results (Table 3) BLEU-2 follows similar trend as demonstrated by BLEU-1 for all test cases with slightly lower values reported for *Trident* model for longer N−Step > 10) ahead sequence.

BLEU-3 and BLEU-4 scores are configured to yield a cumulative score, which is a weighted geometric mean of individual n-gram scores. For instance cumulative BLEU-3 uses (0.33,0.33,0.33,0) and BLEU-4 uses (0.25,0.25,0.25,0.25) as scale weight for 1-gram to 4-gram individual scores. This allows for meaningful scores to capture longer in order token sequences. The in-sample results in Table 3 show *LSTM E-D* models in general yield higher scores than *Trident* models. Even though curriculum learning results lower (approx. < 3%) BLEU scores than Teacher forcing for both the models, but it is able to achieve this with a lower number of Epochs. Therefore, curriculum learning does offer learning at a faster rate, and further improvement is possible. Curriculum learning in particular does seem to help (i.e., yield higher accuracy) for *Trident* model more than it does *LSTM E-D* (approximately by factor of 1.06).

**Figure 18:**
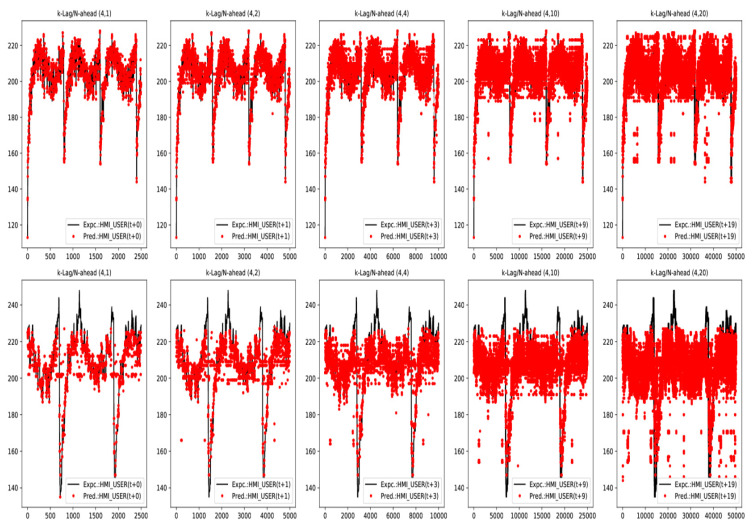
*LSTM E-D* Model—Expected(solid line) vs. Predicted(red dots) Operator response traces for Teacher Forcing test case from In-Sample (top row) & Out-of-Sample (bottom row) data set test under K-Lag=[4] and N-Step=[1,2,4,10,20] runs (in left to right order).

**Figure 19:**
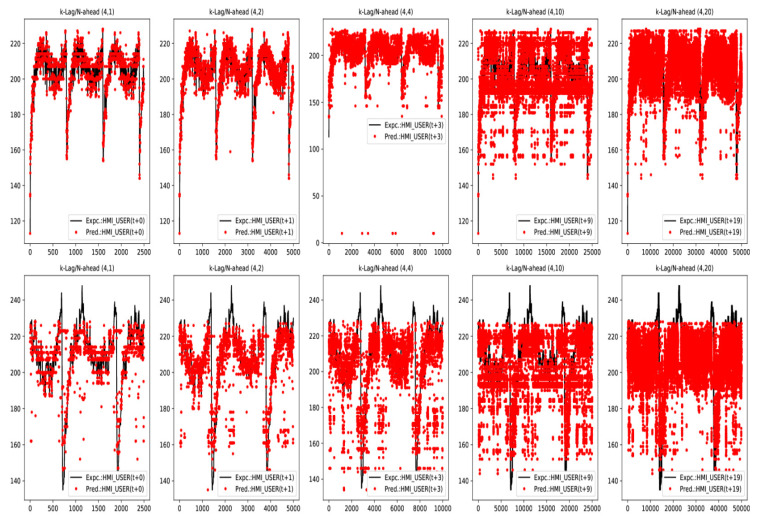
*Trident* Model—Expected(solid line) vs. Predicted(red dots) Operator response traces for Teacher Forcing test case from In-Sample (top row) & Out-of-Sample (bottom row) data sets test under K-Lag=[4] and N-Step=[1,2,4,10,20] runs (in left to right order).

In brevity only a few expected vs. predicted output traces for in-sample and out-of-sample data sets for *LSTM E-D* and *Trident* models under Teacher Forcing case have been included in Figure 18 and Figure 19, respectively. These traces are representative of a general trend of a progressive drop in prediction accuracy as N−Step ahead forecast range increases for a given model trained at a fixed K−Lag value (K-Lag=4). However, as K−Lag is increased, accuracy for longer forecast does improve as evident from results in Table 3 and Table 4.

Machine Translation Accuracy Summary:

- Accuracy(hard) measure for *LSTM E-D* is higher approximately (25%) compared to custom design *Trident* model.
- Accuracy(hard) measure for *LSTM E-D* is lower (approx. < 20%) for curriculum learning than for Teacher Forcing, but former is achieved via faster learning (Epochs=164 vs. Epochs=81).
- BLEU scores generally validate Accuracy(hard) measure, that is, higher Accuracy(hard) value will yield higher BLEU n-gram scores.
- Curriculum learning seems to help *Trident* model more (approximately by factor of 1.06) than it does to *LSTM E-D* model.

Lastly, looking at the training and validation accuracy values obtained during model training (Table 3) shows that—*Trident* model achieves higher validation accuracy than LSTM E-D model. This indicates that *Trident* is less prone to over-fitting and can further be improved by adding more convolution layers.

## 7. Conclusions and Future Work

### 7.1. Conclusions

The broader implication of the proposed language translation modeling approach for the modern control room and transportation industries is to develop a non-intrusive detection of human-in-the-loop (HITL) error precursors and real-time monitoring of operator situational awareness. It may be achieved by capturing and modeling patterns of interaction between the human operator and machine as represented by HMI visual feedback states.

This work demonstrates transforming the HMI state time-series patterns into a language translation problem to position the EYE-on-HMI framework [5] to take advantage of current state-of-the-art NLP models. A custom design of a 1D CNN model (*Trident*) is utilized, which takes into account design features from other state-of-the-art CNN and dot product attention based models (e.g., Transformer [26], etc.). *Trident* model is compared in performance to standard seq2seq LSTM based encoder-decoder design model with attention layer. In addition two NLP model training regimes: Teacher forcing and Curriculum learning based on related research works was also used to train and evaluate the models.

The results indicate the custom 1D CNN model performed (*Trident*) at par if not any less in terms of translation accuracy compared to existing standard seq2seq recurrent LSTM encode-decoder model for both in-sample and out-of-sample data sets. This being an initial design of *Trident*, it shows further room for improvement, as seen in the higher validation accuracy (Table 3). We demonstrate that *Trident* offers parallelism in design that does reasonably well given its simplicity and ability to scale up to to include additional HMI features.

### 7.2. Future Work

A potential limitation of the current work is that it currently addresses HMI state modeling to learn and predict HMI and operator action patterns only. This ability may further be extended to detect anomalous HITL error precursors as a means to monitor SA. Moreover, the proposed SA monitoring approach may even increase nuisance false alarms which, may defeat the purpose of having this system owing to complacent response to actual alarm situations. Such limitations will require future investigation utilizing Naturalistic Driving data for modeling and validation techniques (discussed previously in the background Section 2.2).

Immediate future work entails further improving the CNN based model designs that can scale-up for use modeling industrial control room HMIs. It will require optimizing the models for multi-feature complex sequences using lesser computational resources and training times comparable to current state-of-the-art NLP models. 

## Figures and Tables

**Table 1:** Model Parameter Nomenclatures.

Parameter	Description
(k) or (K)	Fixed time-series window of size *k*, containing *k*-lagged samples from past time steps T=t−k … t−1.
(n) or (N)	Fixed time-series window of size *n*, containing *n*-ahead samples expected in future time steps T=t+0 … t+n−1.
[X], [y]	[X]=<X1,X2> represents an HMI DES event, containing two one-hot encoded feature vectors: X1—HMI indication value vector, X2—User input value vector. Where, each $X2,X2\;\in \;R^{D}$, *D* is the fixed dimensionality (dictionary size) of HMI DES event space (currently set to 255) including the <start> token [y]=<y1,y2> is same in construction as [X], but is used to denote the expected HMI DES event.
${\left\{X\right\}}^{\left(k\right)}$	*k*-lagged sequence of HMI indication (X1) and user input (X2) events containing samples of feature vectors used as model training input pattern.${\left\{X\right\}}^{\left(k\right)}$ = $\{\left(X1_{t-k},X2_{t-k}\right)$, …,$\left(X1_{t-1},X2_{t-1})\right\}$ is a DES HMI state vector of $L_{1}$.
${\left\{y\right\}}^{\left(n\right)}$ ${\left\{\hat{y}\right\}}^{\left(n\right)}$	*n*-step ahead sequence of HMI indication (y1) and user input (y2) events containing samples of feature vector values used as model training target output pattern (ground truth). ${\left\{y\right\}}^{\left(n\right)}$ = $\{\left(y1_{t},y2_{t}\right)$, …,$\left(y1_{t+n-1},y2_{t+n-1})\right\}$. ${\left\{\hat{y}\right\}}^{\left(n\right)}$ is the expected output event sequence or translated HMI DES state vector of $L_{2}$.

**Table 2:** Persistence Score *p-Lag* RMSEp.

n-Lag	*RMSEp*	*RMSEp* (*roll.Win.*)
1	3.75	3.75
2	5.69	3.56
10	9.48	6.62
20	11.08	7.21
50	14.16	9.61

**Table 3:** Seq2Seq Encoder-Decoder Model In-Sample Training Data Set Performance Results.

Test Cases/Metrics	k-Lag	n-Step	RMSEp (roll.Win)	Epocs	RMSErw (rollWin)	Accuracy (Hard)	Accuracy (Tolerance)	BLEU 1	BLEU 2	BLEU 3	BLEU 4	TRN (acc)	VAL (acc)
	4	1	3.75	158	1.153	79.76%	99.88%	1	0	0	0	69.10%	32.10%
	4	20	7.21	164	3.529	72.25%	99.36%	1	0.98	0.92	0.77	85.90%	25.40%
**LSTM E-D**	8	1	3.75	188	4.98	67.04%	98.90%	1	0	0	0	94.20%	18.20%
**Teacher Forcing**	8	20	7.21	135	1.925	93.81%	99.74%	1	1	1	0.99	99.40%	11.00%
	16	20	7.21	149	0.019	99.88%	100.00%	1	1	1	1	99.90%	10.60%
	32	20	7.21	148	**0**	**100.00%**	**100.00%**	1	1	1	1	**100.00%**	9.50%
	4	1	3.75	81	1.262	79.24%	99.76%	1	0	0	0	70.30%	31.40%
	4	20	7.21	81	7.224	38.69%	97.53%	0.99	0.93	0.89	0.79	64.50%	9.00%
**LSTM E-D**	8	1	3.75	81	0.314	95.48%	**100.00%**	1	0	0	0	92.90%	26.10%
**Curriculum Learning**	8	20	7.21	81	4.401	56.03%	98.99%	0.99	0.97	0.94	0.87	93.30%	7.30%
	16	20	7.21	81	2.242	67.44%	99.62%	0.99	0.98	0.96	0.92	99.90%	**6.70%**
	32	20	7.21	81	2.5	65.65%	99.58%	0.99	0.98	0.96	0.91	99.40%	7.10%
	4	1	3.75	41	1.608	67.08%	99.76%	1	0	0	0	99.20%	**83.40%**
	4	20	7.21	421	7.165	46.72%	97.71%	1	0.96	0.94	0.89	95.20%	20.30%
**Trident**	8	1	3.75	41	1.711	66.64%	99.52%	1	0	0	0	99.50%	80.80%
**Teacher Forcing**	8	20	7.21	421	3.559	79.24%	99.37%	1	0.98	0.97	0.94	95.20%	15.70%
	16	20	7.21	421	70.872	41.11%	84.50%	0.85	0.74	0.71	0.62	98.60%	16.00%
	32	20	7.21	421	**90.354**	**28.79%**	**75.43%**	0.75	0.62	0.59	0.49	99.10%	18.50%
	4	1	3.75	81	2.238	54.80%	99.84%	1	0	0	0	85.70%	71.90%
	4	20	7.21	81	18.128	28.75%	95.18%	0.95	0.84	0.78	0.64	**49.70%**	15.20%
**Trident**	8	1	3.75	81	1.902	56.92%	99.88%	1	0	0	0	86.90%	67.10%
**Curriculum Learning**	8	20	7.21	81	13.634	68.87%	98.36%	0.98	0.91	0.86	0.76	86.10%	12.50%
	16	20	7.21	81	16.424	74.36%	97.95%	0.97	0.92	0.88	0.8	91.10%	10.00%
	32	20	7.21	81	15.051	65.22%	98.14%	0.97	0.91	0.87	0.78	92.80%	10.40%

**Table 4:** Seq2Seq Encoder-Decoder Model Out-of-Sample Test Data Set Performance Results.

Test Cases/Metrics	k-Lag	n-Step	RMSEp (roll.Win)	Epocs	RMSErw (rollWin)	Accuracy (hard)	Accuracy (Tolerance)	BLEU 1	BLEU 2	BLEU 3	BLEU 4	TRN (acc)	VAL (acc)
	16	4	4.32	0	**10.72**	16.05%	90.14%	0.99	0.75	0.51	0.19	0	0
	16	10	6.62	0	11.544	12.44%	89.27%	0.99	0.78	0.7	0.44	0	0
**LSTM E-D**	16	20	7.21	0	12.236	11.47%	90.38%	0.98	0.79	0.73	0.54	0	0
**Teacher Forcing**	32	4	4.32	0	11.101	14.59%	90.11%	0.99	0.75	0.51	0.18	0	0
	32	10	6.62	0	12.103	12.20%	89.18%	0.99	0.77	0.68	0.41	0	0
	32	20	7.21	0	12.127	11.40%	90.63%	0.98	0.79	0.73	0.54	0	0
	16	4	4.32	0	10.826	**17.47%**	90.66%	1	0.77	0.58	0.26	0	0
	16	10	6.62	0	11.422	14.36%	89.89%	0.98	0.83	0.79	0.64	0	0
**LSTM E-D**	16	20	7.21	0	12.044	11.57%	89.68%	0.98	0.82	0.78	0.63	0	0
**Curriculum Learning**	32	4	4.32	0	11.156	14.35%	89.78%	0.99	0.81	0.62	0.31	0	0
	32	10	6.62	0	11.359	13.20%	**91.27%**	0.99	0.81	0.76	0.56	0	0
	32	20	7.21	0	12.426	11.55%	90.11%	0.98	0.81	0.77	0.62	0	0
	16	4	4.32	0	11.693	11.66%	88.68%	0.99	0.66	0.39	0.12	0	0
	16	10	6.62	0	17.119	11.25%	88.31%	0.96	0.68	0.58	0.31	0	0
**Trident**	16	20	7.21	0	105.867	8.96%	63.64%	0.68	0.45	0.41	0.27	0	0
**Teacher Forcing**	32	4	4.32	0	16.101	10.37%	86.86%	0.97	0.7	0.46	0.18	0	0
	32	10	6.62	0	12.948	11.40%	89.60%	0.98	0.73	0.65	0.41	0	0
	32	20	7.21	0	**112.988**	**6.18%**	**55.60%**	0.63	0.43	0.4	0.29	0	0
	16	4	4.32	0	11.068	10.00%	89.91%	0.99	0.84	0.68	0.37	0	0
	16	10	6.62	0	15.302	10.29%	84.66%	0.9	0.67	0.6	0.38	0	0
**Trident**	16	20	7.21	0	34.656	9.91%	84.31%	0.9	0.67	0.62	0.45	0	0
**Curriculum Learning**	32	4	4.32	0	15.779	10.32%	84.65%	0.92	0.75	0.61	0.39	0	0
	32	10	6.62	0	14.224	10.64%	87.36%	0.95	0.72	0.66	0.47	0	0
	32	20	7.21	0	33.522	10.40%	87.50%	0.91	0.69	0.65	0.49	0	0
